# Diagnostic value of 5 serum biomarkers for hepatocellular carcinoma with different epidemiological backgrounds: A large-scale, retrospective study

**DOI:** 10.20892/j.issn.2095-3941.2020.0207

**Published:** 2021-02-15

**Authors:** Dongming Liu, Yi Luo, Lu Chen, Liwei Chen, Duo Zuo, Yueguo Li, Xiaofang Zhang, Jing Wu, Qing Xi, Guangtao Li, Lisha Qi, Xiaofen Yue, Xiehua Zhang, Zhuoyu Sun, Ning Zhang, Tianqiang Song, Wei Lu, Hua Guo

**Affiliations:** 1 Department of Hepatobiliary, Liver Cancer Research Center for Prevention and Therapy; 2 Department of Tumor Cell Biology; 3 Clinical Laboratory, Tianjin Medical University Cancer Institute and Hospital, National Clinical Research Center for Cancer, Key Laboratory of Cancer Prevention and Therapy, Tianjin, Tianjin’s Clinical Research Center for Cancer, Tianjin 300060, China; 4 Medical Laboratory, Tianjin Medical University General Hospital, Tianjin 300052, China; 5 Clinical Laboratory, Tianjin Third Central Hospital, Tianjin 300170, China; 6 Department of Pathology, Tianjin Medical University Cancer Institute and Hospital, National Clinical Research Center for Cancer, Key Laboratory of Cancer Prevention and Therapy, Tianjin, Tianjin’s Clinical Research Center for Cancer, Tianjin 300060, China; 7 Department of Tianjin Research Institute of Liver Diseases, Tianjin Second People’s Hospital, Tianjin 300192, China; 8 Department of Infectious Diseases, The First Affiliated Hospital of Baotou Medical College, Inner Mongolia University of Science and Technology, Baotou 014010, China; 9 Department of Epidemiology and Biostatistics, School of Public Health, Tianjin Medical University, Tianjin 300070, China; 10 The Center for Translational Cancer Research, Peking University First Hospital, Beijing 100034, China

**Keywords:** Hepatocellular carcinoma, serum, biomarker, AFP, AFU

## Abstract

**Objective:**

Hepatocellular carcinoma (HCC) is a lethal global disease that requires an accurate diagnosis. We assessed the potential of 5 serum biomarkers (AFP, AFU, GGT-II, GPC3, and HGF) in the diagnosis of HCC.

**Methods:**

In this retrospective study, we measured the serum levels of each biomarker using ELISAs in 921 participants, including 298 patients with HCC, 154 patients with chronic hepatitis (CH), 122 patients with liver cirrhosis (LC), and 347 healthy controls from 3 hospitals. Patients negative for hepatitis B surface antigen and hepatitis C antibody (called “NBNC-HCC”) and patients positive for the above indices (called “HBV-HCC and HCV-HCC”) were enrolled. The selected diagnostic model was constructed using a training cohort (*n* = 468), and a validation cohort (*n* = 453) was used to validate our results. Receiver operating characteristic analysis was used to evaluate the diagnostic accuracy.

**Results:**

The α-L-fucosidase (AFU)/α-fetoprotein (AFP) combination was best able to distinguish NBNC-HCC [area under the curve: 0.986 (95% confidence interval: 0.958–0.997), sensitivity: 92.6%, specificity: 98.9%] from healthy controls in the test cohort. For screening populations at risk of developing HCC (CH and LC), the AFP/AFU combination improved the diagnostic specificity for early-stage HCC [area under the curve: 0.776 (0.712–0.831), sensitivity: 52.5%, specificity: 91.6% in the test group]. In all-stage HBV-HCC and HCV-HCC, AFU was also the best candidate biomarker combined with AFP [area under the curve: 0.835 (0.784–0.877), sensitivity 69.1%, specificity: 87.4% in the test group]. All results were verified in the validation group.

**Conclusions:**

The AFP/AFU combination could be used to identify NBNC-HCC from healthy controls and hepatitis-related HCC from at-risk patients.

## Introduction

Hepatocellular carcinoma (HCC) is the third leading cause of cancer death worldwide, accounting for more than 600,000 deaths each year^1–3^. The prognoses of HCC patients are generally poor, and the median survival of patients is only 6–20 months^4,5^. The main reason for the poor prognosis of HCC is the lack of a timely and accurate diagnosis^6,7^. Thus, according to the epidemiological characteristics of HCC, we divided our research protocol into the diagnoses of NBNC-HCC patients (patients who are negative for hepatitis B surface antigen and hepatitis C antibody)^8^ and the diagnosis of hepatitis (such as HBV or HCV)-related HCC patients^9,10^. Although hepatitis-related HCC accounts for the greatest percentage of HCC patients in China, the percentage of NBNC-HCC patients is rapidly increasing^8,11^. Thus, we recruited this type of HCC patient. We also enrolled patients with a history of alcohol use, aflatoxin exposure, or nonalcoholic steatohepatitis as the “healthy controls,” when compared with the NBNC-HCC patients. This study was conducted because it is critical to develop novel assays to identify NBNC-HCC patients, to increase the likelihood of effective treatments.

When diagnosed at an early stage, HCC can be treated with surgery, transplantation, or radiofrequency ablation, which results in a 5-year survival of 40%–70%^12^, whereas the lack of effective treatments for patients diagnosed with mid- or late-stage disease is associated with a dramatic decrease in survival. Despite the low sensitivity, α-fetoprotein (AFP) is a unique serum biomarker for HCC. Unfortunately, the level of AFP may be elevated in patients with nonmalignant chronic liver diseases, including approximately 40% of patients with hepatitis and 30% of patients with cirrhosis^13^. Thus, only approximately 10%–40% of HCC patients are diagnosed at an early stage using the current AFP-based procedures^14^. This limitation restricts the early diagnoses of HBV-HCC and HCV-HCC based on serum AFP levels.

Over the past several years, serum microRNA panels have become a promising approach for diagnosing early-stage HCC. These panels differentiate HCC patients from healthy and at-risk controls, and provide prognostic values for HCC^15^. However, these panels often require the accurate detection of several serum miRNA levels, which may be complicated and costly for HCC screening in large populations. Thus, use of serum protein biomarkers is still a reliable and economic approach for screening HCC in a large population. In the past decade, many studies of serum biomarkers for detecting HCC have been documented^12,16–18^. More studies have been focused on HBV-HCC, with few studies associated with various etiologies, such as hepatitis C virus infection, alcohol-related liver disease, or nonalcoholic steatohepatitis.

Numerous protein serum biomarkers have been suggested for diagnosing HCC, including α-L-fucosidase (AFU), γ-glutamyl transferase isoenzyme II (GGT-II), glypican-3 (GPC3), and hepatocyte growth factor (HGF)^19–22^. AFU is a lysosomal enzyme detected in most mammalian cells, and is related to the degradation of fucose-containing fucoglycoconjugates^23^. The expression of AFU was higher in HCC samples than in healthy controls and in patients with chronic hepatic disease^24^. GPC3 is a component of heparin sulfate proteoglycans^25^. It is highly expressed in HCC cells and tissues^26^. Recent studies reported that GPC3 was examined in HCC cells, but not in benign liver tissues^27^. GGT-II acted as the second candidate serum marker and was shown to have a higher sensitivity and specificity for hepatoma patients. Surprisingly, it was almost undetectable in other chronic liver diseases^28^. Cui et al.^19^ observed a lower sensitivity and specificity of GGT-II of 74% and 82.2%, respectively. However, their findings still showed GGT-II might be a promising supplemental biomarker for HCC diagnosis. HGF is many times dysregulated, playing an essential role in malignant tumors, including HCC^29^. Kim et al.^30^ reported that the combination of serum bFGF and HGF levels might be candidate biomarkers for HCC patients who could benefit from sorafenib therapy. However, limitations such as small sample sizes and single-center designs have prevented their widespread application.

Herein, we evaluated the sensitivity and specificity of these biomarkers in a large-scale, retrospective study to identify a more accurate diagnostic method for NBNC-HCC and hepatitis-related HCC screening in normal populations and at-risk populations. Our results showed that the combination of AFU and AFP protein biomarkers detected NBNC-HCC in the normal population and in hepatitis-related HCC in the at-risk population with stable and reliable cut-off values. Moreover, the combination maintained diagnostic specificity and improved the sensitivity for the detection of NBNC-HCC and hepatitis-related HCC populations, when compared with AFP alone.

## Materials and methods

### Ethical approval

Our experiments on human subjects were in accordance with the ethical standards of the Helsinki Declaration (amended in 2000) of the World Medical Association. In addition, this study was approved by the Ethics Committees of Tianjin Medical University Cancer Institute and Hospital (Approval No. bc2020083). All patients were informed about the study, and gave their consent for participation.

### Study design and patients

A total of 996 subjects who visited the Tianjin Medical University Cancer Institute and Hospital, Tianjin Medical University General Hospital or Tianjin Third Central Hospital between July 2012 and April 2014 were recruited in this study for different cohorts (**Supplementary Figure S1**). Patients with HCC were diagnosed based on ultrasound, computed tomography, or magnetic resonance imaging, and the diagnoses were confirmed histopathologically according to the AASLD guidelines. According to different etiologies, we divided the HCC patients into the hepatitis-related HCC and NBNC-HCC groups. Tumor stage was defined according to the Barcelona Clinic Liver Cancer (BCLC) staging system. For the purpose of this study, we classified tumors with BCLC stage 0 + A as early-stage hepatitis-related HCC and patients who were suffering from chronic hepatitis or liver cirrhosis as at-risk patients. None of the patients underwent any treatment, including surgery, chemotherapy, or radiotherapy, before blood sampling. The diagnosis of cirrhosis was confirmed by liver biopsy and/or clinical, laboratory, and imaging evidence. Chronic hepatitis was defined as chronic necroinflammatory disease of the liver caused by persistent HBV or HCV infection. Healthy controls were used for comparison with NBNC-HCC patients. They were recruited from the Physical Examination Center at Tianjin Medical University Cancer Institute and Hospital, and were eligible if they had no viral hepatitis and no malignant disease. Participants with a history of alcohol use, aflatoxin exposure, or nonalcoholic steatohepatitis also met the criteria for healthy controls. Patients were excluded for the following reasons: (1) 27 patients had primary liver cancer other than HCC, (2) 2 patients had metastatic liver cancer, (3) 11 patients had liver sarcoma or adenocarcinoma, and (4) 35 patients did not have available clinical data. Thus, 468 patients, including 150 HCC patients (123 hepatitis-related HCC patients and 27 NBNC-HCC patients), 82 chronic hepatitis (CH) patients, 61 liver cirrhosis (LC) patients, and 175 healthy controls (HC), were recruited from the three hospitals as the test group between July 2012 and June 2013. When we finished the analysis of the test group, 453 patients who were matched for age and sex with the test group were recruited from the same hospitals as the validation group. The validation cohort was comprised of 121 hepatitis-related HCC patients, 72 CH patients, and 61 LC patients as one subgroup and 27 NBNC-HCC patients and 172 HC patients as another subgroup. The data involving 5 factors and demographic characteristics such as sex and age of patients are listed in **Supplementary Table S1–S5**.

### Statistical analysis

Statistical analyses were performed using SPSS statistical software for Windows, version 25.0 (SPSS, Chicago, IL, USA) and MedCalc, version 18.2.1 (https://www.medcalc.org/). Differences between two independent groups were tested using the Mann-Whitney U test (continuous variables and nonparametric analyses). *P* values < 0.05 were considered to be significant, and all *P* values were two-sided. To assess whether the combination of AFU and AFP was better than either of them alone, a new variable predicted probability (*P*) for HCC was created on the basis of an equation obtained by binary logistic regression:

- all-stage HBV-HCC and HCV-HCC *vs.* CH and LC in the test cohort:
  (*P*): 0.007668*AFP + 0.033718*AFU-1.347426
- all-stage HBV-HCC and HCV-HCC *vs.* CH and LC in the validation cohort
  (*P*): 0.001227*AFP + 0.017566*AFU-0.957458
- early-stage HBV-HCC and HCV-HCC *vs.* CH and LC in the test cohort
  (*P*): 0.005890*AFP + 0.018753*AFU-1.557863
- early-stage HBV-HCC and HCV-HCC *vs.* CH and LC in the validation cohort
  (*P*): 0.000921*AFP + 0.011742*AFU-1.166897
- NBNC-HCC *vs.* HC in the test cohort
  (*P*): 0.059672*AFP + 0.403175*AFU-8.669705
- NBNC-HCC *vs.* HC in the validation cohort
  (*P*): 0.047177*AFP + 0.211019*AFU-5.707287

### Nomogram for the hepatitis-related HCC and NBNC-HCC populations

A nomogram was formulated based on the results of logistic regression analyses and by using the rms package of R, version 3.0 (http://www.r-project.org/). The nomogram was based on proportionally converting each regression coefficient in multivariate logistic regression to a total points scale. For the diagnosis of HCC based on the model, the total score for each participant was calculated with the nomogram. We could preliminarily predict the likelihood of a participant suffering from HCC based on the probability.

### Blood samples

Blood samples were obtained by peripheral venous puncture before any surgical or chemotherapeutic treatment. After clotting and within 1 h of collection, the blood samples were centrifuged at 3,000 × *g* for 5 min, and serum aliquots were stored at –80 °C until analysis.

### Serum tumor marker detection

The AFP, AFU, GGT-II, GPC3, and HGF serum levels were analyzed according to the manufacturer’s instructions using ELISA kits (Cusabio, Wuhan, China and eBioscience, San Diego, CA, USA). All assays were performed in duplicate.

### Immunohistochemistry (IHC) staining

IHC staining was used to examine the expression levels of AFU in paraffin-embedded samples of HCC tissues according to previously described methods^31^. An anti-AFU (FUCA2) antibody was purchased from Bioss (bs-16192R, 1:200; Bioss, Woburn, MA, USA). The IHC score was used to evaluate the correlation between AFU expression and overall survival (OS) and disease-free survival (DFS) of HCC patients.

### Bioinformatic analysis

Correlation between AFU or AFP/AFU combination expression and overall/DFS in HCC patients was based on the Kaplan-Meier method (http://kmplot.com/analysis/). The threshold of significance was set at *P* < 0.05.

## Results

### The serum levels of AFP, AFU, GPC3, GGT-II, and HGF in the test group

**Figure 1:**
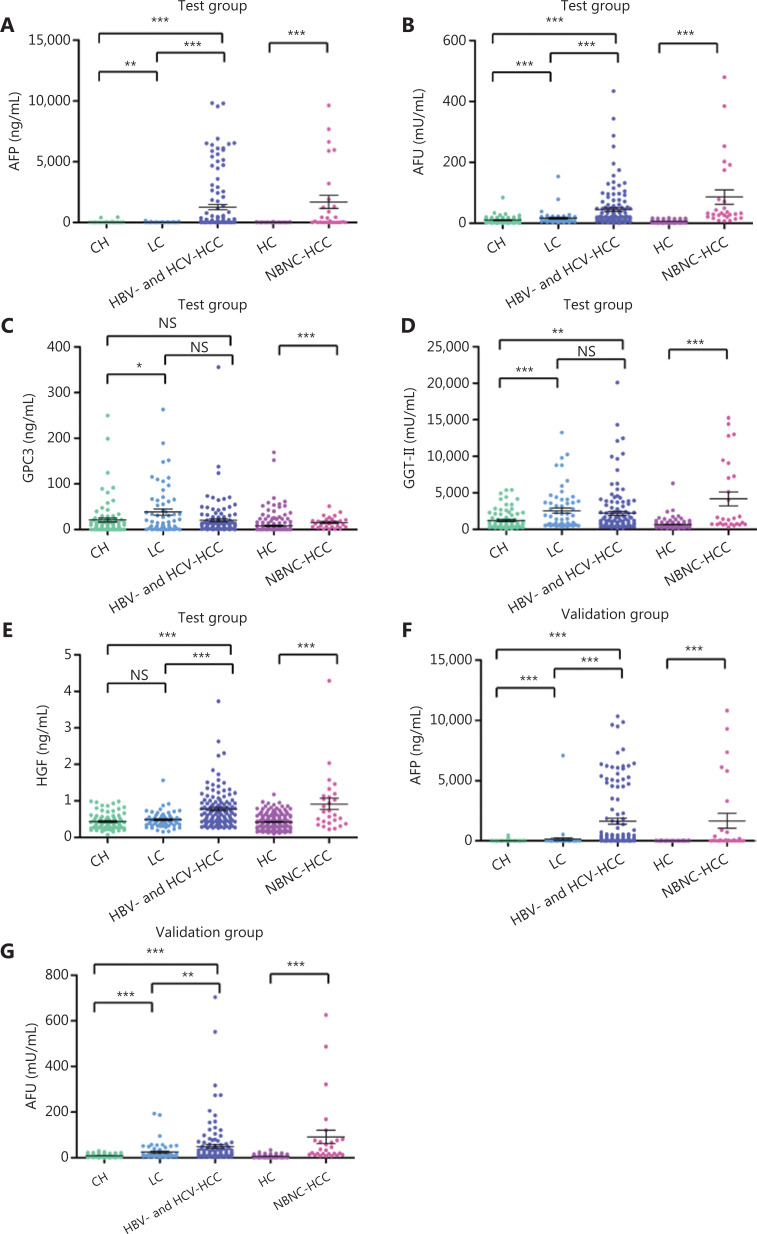
The median plasma levels of AFP (A), AFU (B), GPC3 (C), GGT-II (D), and HGF (E) in the test cohort and AFP (F) and AFU (G) in the validation cohort. HC, healthy controls; CH, chronic hepatitis; LC, liver cirrhosis; HCC, hepatocellular carcinoma. *P < 0.05; **P < 0.01; ***P < 0.001; *P* > 0.05 means no significance (NS).

In the test cohort, all 463 patients were tested for serum levels of AFP, AFU, GPC3, GGT-II, and HGF. The median plasma levels of all 5 tumor markers were found to be significantly higher in the NBNC-HCC subgroup than in the healthy controls (**Figure 1A–1E**). In the HBV-HCC and HCV-HCC subgroups, the levels of AFU, GPC3, and GGT-II were significantly higher in the LC group than in the CH group (**Figure 1B–1D**), suggesting that elevated levels of these three biomarkers may be associated with the progression of hepatitis to liver cirrhosis. The HBV-HCC and HCV-HCC patient median plasma levels of AFP, AFU, and HGF were found to be significantly higher than those of the CH and LC patients (**Figure 1A, 1B, and 1E**), indicating that a high expression of these biomarkers was associated with the progression of liver disease. Generally, a high level of these 3 candidate markers was associated with the onset of HBV-HCC and HCV-HCC.

### The combination of AFP and AFU had high accuracy in the detection of NBNC-HCC

**Figure 2:**
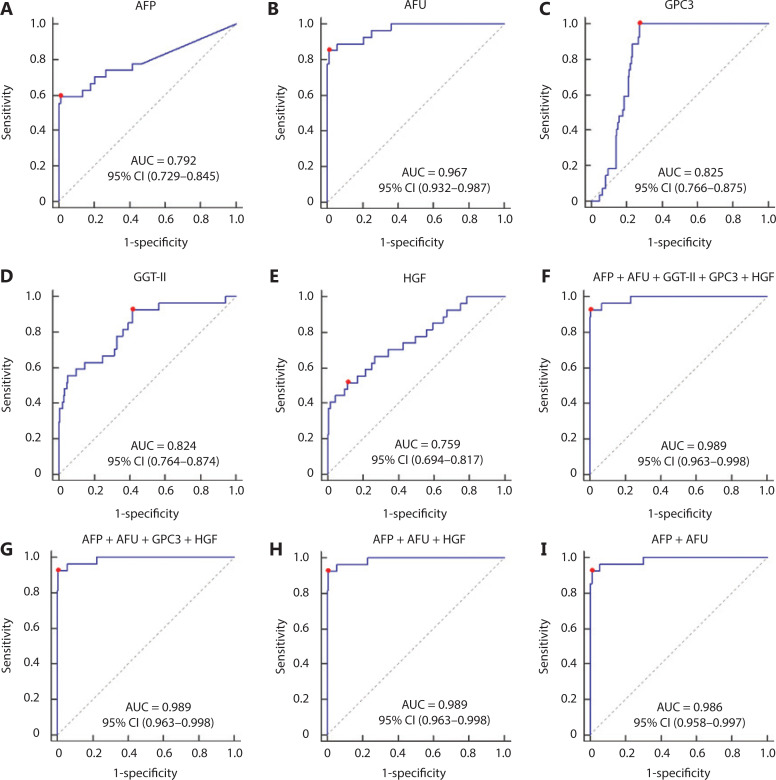
The receiver operating characteristic curve of AFP (A), AFU (B), GPC3 (C), GGT-II (D), HGF (E), AFP + AFU + GGT-II + GPC3 + HGF (F), AFP + AFU + GPC3 + HGF (G), AFP + AFU + HGF (H), and AFP + AFU (I) in the detection of the NBNC-HCC test group. The sensitivity and specificity represented by the red dots are shown in detail (lower). (A). AFP sensitivity: 59.3% and specificity: 98.9%; (B). AFU sensitivity: 85.2% and specificity: 98.9%; (C). GPC3 sensitivity: 100.0% and specificity: 72.6%; (D). GGT-II sensitivity: 92.6% and specificity: 58.3%; (E). HGF sensitivity: 51.9% and specificity: 88.6%; (F). AFP + AFU + GPC3 + GGT-II + HGF sensitivity: 92.6% and specificity: 98.9%; (G). AFP + AFU + GPC3 + HGF sensitivity: 92.6% and specificity: 99.4%; (H). AFP + AFU + HGF sensitivity: 92.6% and specificity: 99.4%; (I). AFP + AFU sensitivity: 92.6% and specificity: 98.9%.

The area under the curve (AUC) values of AFP, AFU, GPC3, GGT-II, and HGF were 0.792, 0.967, 0.825, 0.824, and 0.759, respectively (**Figure 2A–2E**). Each serum biomarker could be a candidate serum biomarker combined with AFP in diagnosing NBNC-HCC. Thus, we determined the different values of the receiver operating characteristic (ROC) curve with various combinations of serum biomarkers (AFP, AFU, GPC3, GGT-II, and HGF). The 5 biomarker combinations performed well (AUC: 0.989, sensitivity: 92.6%, specificity: 98.9%) (**Figure 2F**). The best 4/3/2 biomarker combinations had a similar AUC, sensitivity, and specificity compared with the 5 biomarker combination (AUC: 0.989, sensitivity: 92.6%, specificity: 99.4%; AUC: 0.989, sensitivity: 92.6%, specificity: 99.4%; AUC: 0.986, sensitivity: 92.6%, specificity: 98.9%, respectively) (**Figure 2G–2I**). The combination results of the remaining serum indicators are shown in **Supplementary Figure S2–S4**. After combining various factors (such as AUC, sensitivity, and specificity), we chose the combination of AFP and AFU as the diagnostic combination for NBNC-HCC. The predictive values and likelihood ratios for AFU and AFP in the diagnosis of NBNC-HCC are shown in **Table 1**. The combination improved the sensitivity of AFP in diagnosing NBNC-HCC, while the specificity was relatively unchanged.

**Table 1:** Results for the measurement of serum AFU, AFP, or both, in the diagnosis of NBNC-HCC

	Test								Validation							
	AUC (95% CI)	Sensitivity (%)	Specificity (%)	PPV (%)	NPV (%)	Positive LR	Negative LR	*P* value	AUC (95% CI)	Sensitivity (%)	Specificity (%)	PPV (%)	NPV (%)	Positive LR	Negative LR	*P* value
NBNC-HCC *vs*. HC (results for measurement of AFU, AFP, or both in the diagnosis of NBNC-HCC)																
AFP	0.792 (0.729–0.845)	59.3%	98.9%	88.9%	94.0%	51.85	0.41	<0.001	0.707 (0.639–0.769)	51.9%	97.7%	77.8%	92.8%	22.25	0.49	0.002
AFU	0.967 (0.932–0.987)	85.2%	98.9%	92.0%	97.7%	74.54	0.15	<0.001	0.948 (0.907–0.974)	74.1%	96.5%	76.9%	96.0%	21.23	0.27	<0.001
AFP + AFU	0.986 (0.958–0.997)	92.6%	98.9%	92.6%	98.9%	81.02	0.075	<0.001	0.969 (0.934–0.988)	88.9%	94.8%	72.7%	98.2%	16.99	0.12	<0.001

The diagnostic cutoff values of serum AFP and AFU were 43.23 ng/mL and 16.75 mU/mL, respectively.

**Figure 3:**
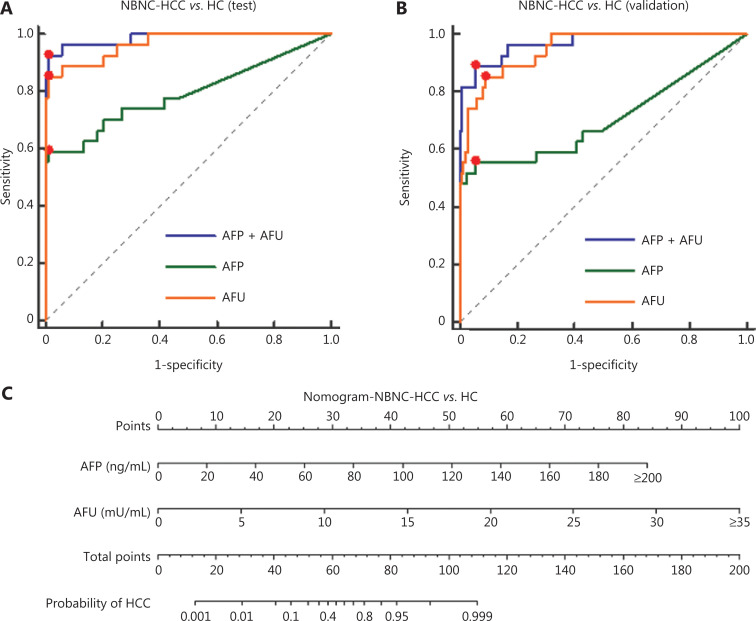
Diagnostic outcomes and nomograms for the combination of serum AFP and AFU in the diagnosis of NBNC-HCC. (A). Receiver operating characteristic curves (ROCs) for AFU, AFP, or both for all patients with NBNC-HCC *vs*. HC in the test cohort. (B). ROC curves for AFU, AFP, or both for all patients with NBNC-HCC *vs.* HC in the validation cohort. (C). Nomogram of the combined AFP/AFU in diagnosing NBNC-HCC.

According to the stable cutoff value of AFP and AFU in detecting NBNC-HCC, we verified the results of the test cohort in the validation cohort. First, the trends of AFP and AFU concentrations in healthy controls and NBNC-HCC patients were consistent with those in the test cohort (**Figure 1F and 1G**). Furthermore, the AUC, sensitivity, specificity, PPV (positive predictive value), NPV (negative predictive value), positive LR (likelihood ratio), and negative LR of AFP, AFU, and their combinations were similar to those in the test cohort at the optimum cut-off value (**Supplementary Figure S5 and Table 1**). The AUC of the combination was better than any other single biomarker (only AFP or AFU) of NBNC-HCC in the test and validation groups (**Figure 3A and 3B**). We used a nomogram model for the clinical application of these 2 serum markers (**Figure 3C**). For example, if the AFP and AFU values of a “healthy person” (including individuals with a history of alcohol, aflatoxin exposure, or nonalcoholic steatohepatitis) were 50 ng/mL and 20 mU/mL, respectively, then based on the nomogram model, the probability of this participant developing NBNC-HCC was nearly 90%.

### The combined AFP/AFU panel showed an improvement in the diagnostic sensitivity for the detection of all-stage and early-stage hepatitis-related HCC

**Figure 4:**
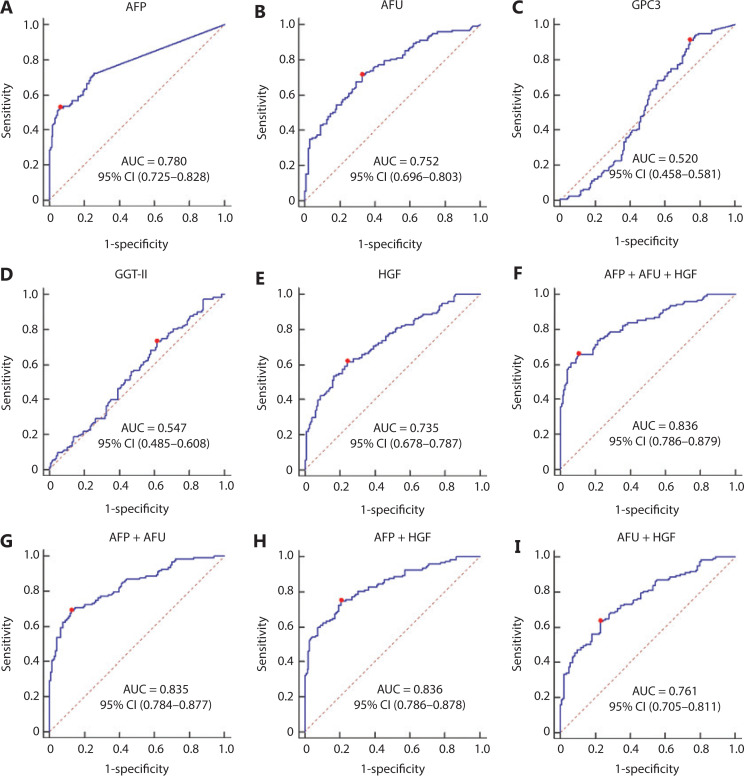
The receiver operating characteristic curves of AFP (A), AFU (B), GPC3 (C), GGT-II (D), and HGF (E), AFP + AFU + HGF (F), AFP + AFU (G), AFP + HGF (H), AFU + HGF (I) in the detection of all-stage HBV-HCC and HCV-HCC of the test group. The sensitivity and specificity represented by the red dots are shown in detail (lower). (A). AFP sensitivity: 52.8% and specificity: 93.7%; (B). AFU sensitivity: 71.5% and specificity: 67.1%; (C). GPC3 sensitivity: 91.1% and specificity: 25.9%; (D). GGT-II sensitivity: 73.2% and specificity: 38.5%; (E). HGF sensitivity: 61.8% and specificity: 75.5%; (F). AFP + AFU + HGF sensitivity: 65.9% and specificity: 89.5%; (G). AFP + AFU sensitivity: 69.1% and specificity: 87.4%; (H). AFP + HGF sensitivity: 74.8% and specificity: 79.0%; (I). AFU + HGF sensitivity: 63.4% and specificity: 76.9%.

**Figure 5:**
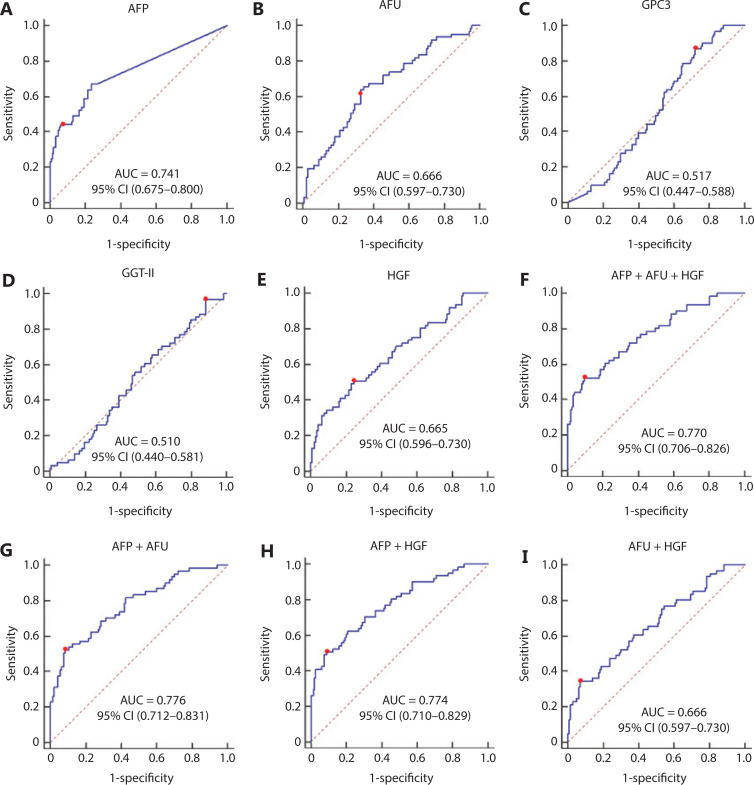
The receiver operating characteristic curves of AFP (A), AFU (B), GPC3 (C), GGT-II (D), and HGF (E), AFP + AFU + HGF (F), AFP + AFU (G), AFP + HGF (H), and AFU + HGF (I) in the detection of early-stage HBV-HCC and HCV-HCC of the test group. The sensitivity and specificity represented by the red dots are shown in detail (lower). (A). AFP sensitivity: 44.3% and specificity: 93.7%; (B). AFU sensitivity: 63.9% and specificity: 67.1%; (C). GPC3 sensitivity: 86.9% and specificity: 28.0%; (D). GGT-II sensitivity: 96.7% and specificity: 11.9%; (E). HGF sensitivity: 50.8% and specificity: 75.5%; (F). AFP + AFU + HGF sensitivity: 52.5% and specificity: 90.2%; (G). AFP + AFU sensitivity: 52.5% and specificity: 91.6%; (H). AFP + HGF sensitivity: 50.8% and specificity: 90.9%; (I). AFU + HGF sensitivity: 34.4% and specificity: 93.0%.

The AUC values of AFP, AFU, GPC3, GGT-II, and HGF in the all-stage HBV-HCC and HCV-HCC groups were 0.780, 0.752, 0.520, 0.547, and 0.735, respectively (**Figure 4A–4E**). Because there was no significance between GGT-II and GPC3 in detecting all-stage HBV-HCC and HCV-HCC, we chose AFP, AFU, and HGF in a combination model (**Figure 4F–4I**). The diagnostic performance of the serum biomarkers in different subgroups was further evaluated. Among these combinations, the AFP/AFU panel outperformed the others and exhibited a greater diagnostic sensitivity and specificity for the differentiation of all-stage HBV-HCC and HCV-HCC patients from CH and LC patients [AUC: 0.835 (0.784–0.877), sensitivity: 69.1%, specificity: 87.4%] (**Figure 4G**). The diagnostic values of serum AFP and AFU were 42.34 ng/mL and 13.94 mU/mL, respectively. Regarding early stage HBV-HCC and HCV-HCC, the AUC values of AFP, AFU, GPC3, GGT-II, and HGF were 0.741, 0.666, 0.517, 0.510, and 0.665, respectively (**Figure 5A–5E**). We observed similar results in this test cohort with the all-stage HBV-HCC and HCV-HCC groups. AFP, AFU, and HGF were selected for the combination model (**Figure 5F–5I**). The AFP/AFU combination was also notable for early-stage HBV-HCC and HCV-HCC [AUC: 0.776 (0.712–0.831), sensitivity: 52.5%, specificity: 91.6%] in the test cohort (**Figure 5G**). In summary, the AFP/AFU panel improved the diagnostic sensitivity without a loss of specificity in the detection of all-stage HBV-HCC and HCV-HCC (**Table 2**: AFP *vs.* AFP + AFU: sensitivity 52.8% *vs.* 69.1%, specificity 93.7% *vs*. 87.4%) and early-stage HBV-HCC and HCV-HCC (**Table 2**: AFP *vs*. AFP + AFU, sensitivity: 44.3% *vs.* 52.5%, specificity: 93.7% *vs*. 91.6%) among at-risk patients.

**Table 2:** Results for measurement of serum AFU, AFP, or both, in the diagnosis of HBV-HCC and HCV-HCC

	Test								Validation							
	AUC (95% CI)	Sensitivity (%)	Specificity (%)	PPV (%)	NPV (%)	Positive LR	Negative LR	*P* value	AUC (95% CI)	Sensitivity (%)	Specificity (%)	PPV (%)	NPV (%)	Positive LR	Negative LR	*P* value
Hepatitis-HCC *vs*. CH and LC (results for the measurement of AFU, AFP, or both in diagnosis of hepatitis-HCC)																
AFP	0.780 (0.725–0.828)	52.8%	93.7%	87.8%	69.8%	8.40	0.50	<0.001	0.809 (0.755–0.855)	62.8%	90.2%	85.4%	72.7%	6.41	0.41	<0.001
AFU	0.752 (0.696–0.803)	71.5%	67.1%	65.2%	73.3%	2.18	0.42	<0.001	0.727 (0.668–0.781)	69.4%	65.4%	64.6%	70.2%	2.00	0.47	<0.001
AFP + AFU	0.835 (0.784–0.877)	69.1%	87.4%	82.5%	76.7%	5.49	0.35	<0.001	0.841 (0.790–0.884)	71.9%	86.5%	82.9%	77.2%	5.31	0.32	<0.001
Early hepatitis-HCC *vs*. CH and LC (results for measurement of AFU, AFP, or both in diagnosis of early hepatitis-HCC)																
AFP	0.741 (0.675–0.800)	44.3%	93.7%	75.0%	79.8%	7.03	0.59	<0.001	0.758 (0.693–0.816)	52.2%	90.2%	73.5%	78.4%	5.33	0.53	<0.001
AFU	0.666 (0.597–0.730)	63.9%	67.1%	45.3%	81.4%	1.94	0.54	<0.001	0.671 (0.602–0.736)	56.5%	65.4%	45.9%	74.4%	1.63	0.67	<0.001
AFP + AFU	0.776 (0.712–0.831)	52.5%	91.6%	72.7%	81.9%	6.25	0.52	<0.001	0.791 (0.728–0.845)	75.4%	73.7%	59.8%	85.2%	2.86	0.33	<0.001

The diagnostic cutoff values of serum AFP and AFU were 42.34 ng/mL and 13.94 mU/mL, respectively.

**Figure 6:**
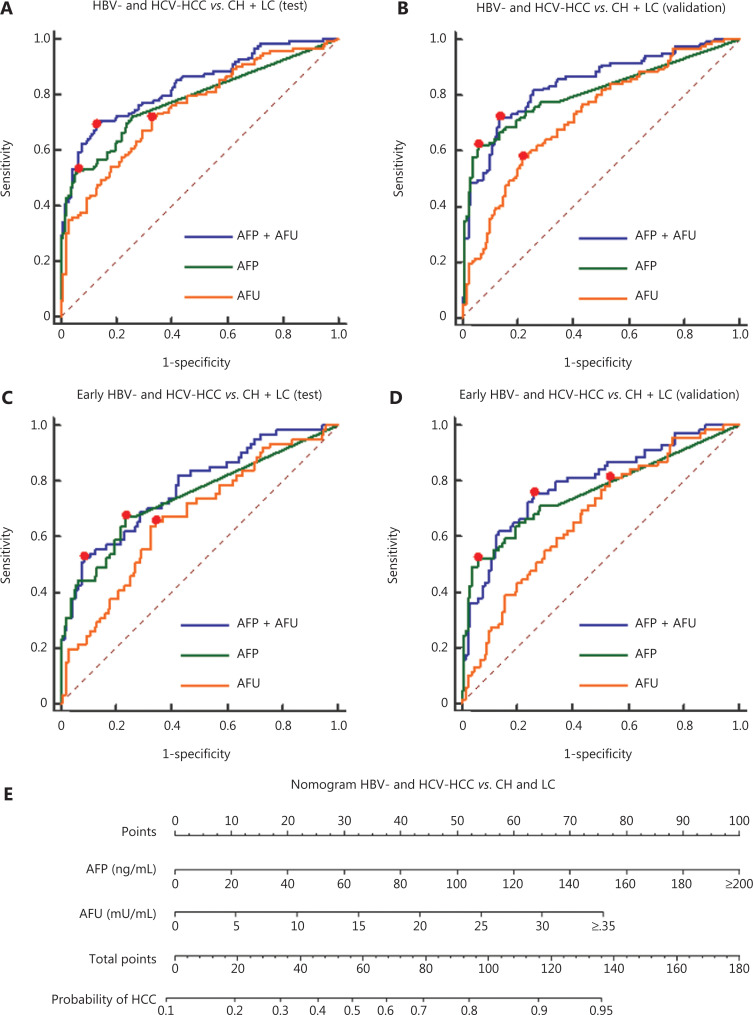
Diagnostic outcomes and nomogram for the combination of serum AFP and AFU of all-stage and early stage hepatitis-related hepatocellular carcinoma (HCC). (A). Receiver operating characteristic curves (ROCs) for AFU, AFP, or both for all patients with all-stage HBV-HCC and HCV-HCC *vs*. CH and LC in the test cohort. (B). ROC curves for AFU, AFP, or both for all patients with all-stage HBV-HCC and HCV-HCC *vs*. CH and LC in the validation cohort. (C). ROC curves for AFU, AFP, or both for all patients with early-stage HBV-HCC and HCV-HCC *vs*. CH and LC in the test cohort. (D). ROC curves for AFU, AFP, or both for all patients with early-stage HBV-HCC and HCV-HCC *vs*. CH and LC in the validation cohort. (E). Nomogram of the combined AFP/AFU in diagnosing HBV-HCC and HCV-HCC.

In the validation cohort, the concentrations of AFP and AFU in CH-, LC-, and hepatitis-related HCC patients are shown in **Figure 1F and 1G**. The results were similar to those in the test cohort. The ROC curves of single serum markers and combined serum markers in the validation group are shown in **Supplementary Figure S6** (the results for all-stage HBV-HCC and HCV-HCC are shown in A, B, and E; the results for early-stage HBV-HCC and HCV-HCC are shown in C, D, and F). Compared with the optimum diagnostic cutoff values of AFP and AFU for HBV-HCC and HCV-HCC in the test group, the parameter values in the validation group for all-stage and early-stage hepatitis-related HCC are summarized in **Table 2** [AUC: 0.841 (0.790–0.884), sensitivity: 71.9%, specificity: 86.5% in the validation cohort for all-stage HBV-HCC and HCV-HCC; AUC: 0.791 (0.728–0.845), sensitivity: 75.4%, specificity: 73.7% in the validation cohort for early-stage HBV-HCC and HCV-HCC]. The AUC of the AFP/AFU combination was better than any other single biomarker (only AFP or AFU) of all-stage (**Figure 6A and 6B**) and early-stage (**Figure 6C and 6D**) hepatitis-related HCC in the test and validation groups. We also constructed a nomogram model for the clinical application of these 2 serum markers in HBV-HCC and HCV-HCC (**Figure 6E**). For example, if the AFP and AFU values of an “at-risk person” (such as an individual with HBV or HCV) were 60 ng/mL and 25 mU/mL, respectively, then based on the nomogram model, the probability of this individual developing HBV-HCC and HCV-HCC was nearly 75%.

Overall, the AFP/AFU panel improved the accuracy for diagnosing all-stage and early-stage hepatitis-related HCC compared to any other single marker. Moreover, the inclusion of demographic characteristics assisted in the detection of disease.

### The AFP/AFU combination was effective in predicting the survival of HCC patients

We evaluated the AFU levels in predicting HCC patient prognoses based on a KM plotter database. The results showed that patients with low expression of AFU might have better prognoses (**Supplementary Figure S7A and B**). Thus, we assessed the value of the AFP/AFU combination in forecasting survival of HCC patients. The 5-year overall/DFS of HCC patients with low expression of the AFP/AFU combination was almost 60% and 40%, respectively, while the survival of patients with high expression was approximately 40% and 25%, respectively (**Supplementary Figure S7C and D**). Overall, the results showed that the AFP/AFU combination was effective in predicting HCC prognosis.

We also verified the results of the KM plotter based on our IHC data. First, we found that patients with high AFU levels had worse prognoses (**Supplementary Figure S7E and F**). In addition, the IHC results of the AFP/AFU combination in predicting HCC prognoses were consistent with those in the KM plotter database (**Supplementary Figure S7G and H**). Thus, the AFP and AFU panel was effective in predicting the survival of HCC patients.

## Discussion

There is a consensus that early diagnosis is the key to improving the survival of HCC patients^4^. Several preliminary studies have suggested that serum biomarkers, including AFP, AFU, GGT-II, HGF, and GPC3, may be used for the diagnosis of HCC^19–22^. However, these markers are not currently included in routine clinical assessments because of the lack of large-scale, multicenter clinical investigations.

Over the past 2 decades, infection with hepatitis B virus (HBV) or hepatitis C virus (HCV) has been associated with approximately 85% of worldwide HCC^32^. Due to the promotion of antiviral therapy, the number of patients with other causes of HCC (hepatitis B virus surface antigen-negative and hepatitis C virus antibody-negative or NBNC-HCC) is increasing^8,11^. In our study of 401 subjects (202 in the test cohort and 199 in the validation cohort), the levels of all 5 markers were significantly higher in NBNC-HCC patients than in healthy controls. We therefore further studied the diagnostic capabilities of these 5 markers for NBNC-HCC. It is worth mentioning that the healthy controls in this study only referred to individuals who had not been infected with HBV or HCV. The healthy controls may have suffered from alcohol-related liver disease, nonalcoholic steatohepatitis, or aflatoxin exposure^33^. The combination of AFP and AFU was uniquely associated with the progression of NBNC-HCC (HC to NBNC-HCC). This combination showed promising characteristics as a diagnostic marker for NBNC-HCC. Their diagnostic capability outperformed that of any other serum marker in this study (AUC: 0.986, 95% CI: 0.958–0.997, sensitivity: 92.6%, specificity: 98.9% in the test cohort; AUC: 0.969, 95% CI: 0.934–0.988, sensitivity: 88.9%, specificity: 94.8% in the validation cohort). Considering various factors, such as the incidence of NBNC-HCC, our study is the first large-scale, retrospective analysis of serum biomarkers in NBNC-HCC patients.

In China, the incidence and mortality of hepatitis-related HCC is still high^34^. The HBV-HCC and HCV-HCC patient median plasma levels of AFP, AFU, and HGF were found to be significantly higher than those of CH and LC patients. We showed that the rise of these 3 serum biomarkers may be related to the occurrence of HBV-HCC and HCV-HCC. Thus, we paid particular attention to these 3 serum markers for differentiating HBV-HCC and HCV-HCC patients from at-risk (CH and LC) patients. This differentiation has also been the focus of current research worldwide^35^. In our study, AFU showed promising accuracy in identifying HBV-HCC and HCV-HCC patients from the at-risk population. We found that the combination of AFP and AFU uniquely reflected the progression of HBV-HCC and HCV-HCC (CH to LC to HBV-HCC and HCV-HCC). For all-stage hepatitis-related HCC *vs.* CH and LC, the ROC curves showed that the AFP/AFU combination had an AUC of 0.835 (95% CI: 0.784–0.877), a sensitivity of 69.1%, and a specificity of 87.4%. Our results are comparable with other promising markers, especially in terms of diagnostic sensitivity (e.g., DKK1: 74.8% *vs*. 69.1% in all-stage detection)^12^. Similar results were also shown in early-stage HBV-HCC and HCV-HCC (AUC: 0.776, 95% CI: 0.712–0.831, sensitivity: 52.5%, specificity: 91.6%). Most importantly, the AFP/AFU panel improved the diagnostic sensitivity in the absence of a loss of specificity in the detection of HBV-HCC and HCV-HCC. Notably, this strategy showed an advantage for using an AFP/AFU panel. Our findings are consistent with the results of basic and clinical research studies^23,36,37^. AFU was also considered to be a prognostic and disease recurrence marker and has been shown to be associated with metastasis and reduced overall survival^38^.

Zhang et al.^39^ assessed the diagnostic value for HCC in combination with AFU, AFP, and TK1. They enrolled participants including 116 patients with HCC, 109 patients with benign hepatic diseases (such as hepatitis and liver cirrhosis), and 104 normal subjects. The results showed that the AUC was 0.718 for AFU, 0.832 for AFP, 0.773 for TK1, and 0.900 for the combination of these markers. The results were similar to our data in the detection of HBV-HCC and HCV-HCC (0.780 for AFP, 0.752 for AFU, and 0.835 for the combination). In addition, we showed that the AFP/AFU combination was effective in detecting NBNC-HCC patients. Zhu et al.^40^ found that the AUCs were 0.80, 0.80, and 0.87 for serum AFU, 5′-NT, and AFP, respectively. The correlation of AFU and AFP was significant. However, they did not identify the combination of these markers. In addition, the number of participants was too low (36 for HCC and 36 for healthy controls). Xing et al.^24^ reported that a combination of AFU and AFP (AUC: 0.582) did not improve the diagnostic efficacy compared with AFP (AUC: 0.764) alone for HCC patients. They showed that the majority of HCC patients (85.5%) had chronic HBV and only 13 patients (6.9%) had chronic HCV, so there were some NBNC-HCC patients (7.6%) who were enrolled in the HCC cohort. Based on the etiology of HCC, patients with hepatitis do not progress to NBNC-HCC. This part of NBNC-HCC patients might therefore cause bias in the results. However, patients with benign disease would not evolve to HBV or HCV-related HCC. This phenomenon might lead to a low AUC of AFU and its combination. We enrolled patients with hepatitis or liver cirrhosis as controls of hepatitis-related HCC patients. In addition, healthy controls were used for comparison with NBNC-HCC patients. Thus, the results regarding the AUC of the AFP/AFU combination in our study were more convincing.

## Conclusions

To the best of our knowledge, this is the first report showing the potential of AFU in diagnosing NBNC-HCC and hepatitis-related HCC, based on a study with a large sample size and independent validation. Wang et al.^21^ reported that preoperative serum AFU is a prognostic predictor of HCC based on survival prognosis data. We showed that AFU was a promising diagnostic marker for NBNC-HCC and hepatitis-related HCC, with a high degree of accuracy and clinically applicable cut-off concentrations; AFU could also serve as a reliable second-line marker for the detection of HCC. The AFP/AFU panel had a high degree of accuracy for differentiating NBNC-HCC from healthy controls and hepatitis-related HCC in patients at risk for developing HCC. The assays, which are easy to perform and cost effective, can be translated into a standard protocol for the clinical diagnosis of HCC, which may identify asymptomatic patients early for curative treatments. We are currently conducting a prospective study to confirm the present findings and to determine the potential utility of measuring AFP/AFU levels to monitor therapeutic responses, and for the prognostic diagnosis of HCC.
