# Supplementary material for: Diagnostic value of 5 serum biomarkers for hepatocellular carcinoma with different epidemiological backgrounds: A large-scale, retrospective study
Source: Cancer Biol Med. 2021 Feb 15;18(1):256–70. doi: 10.20892/j.issn.2095-3941.2020.0207 (PMC7877174; doi:10.20892/j.issn.2095-3941.2020.0207)
Supplement: Supplementary file 1 [file cbm-18-256-s001.pdf]

Supplementary materials

Table S1a The information of 347 healthy controls (test group)

Number	AFP (ng/mL)	AFU (mU/mL)	GGT2 (mU/mL)	GPC3 (ng/mL)	HGF (ng/mL)	Gender	Age
**2**	0.000	6.127	515.942	0.285	0.390	Female	43
**4**	0.000	8.380	425.294	0.000	0.338	Female	37
**5**	0.000	6.177	504.611	0.000	0.292	Male	33
**6**	0.000	11.423	512.364	0.000	0.334	Female	31
**8**	0.000	3.335	470.021	0.000	0.253	Female	27
**9**	5.057	7.472	175.415	0.000	0.395	Female	45
**14**	0.000	10.465	345.976	1.373	0.296	Female	31
**18**	1.253	9.153	315.562	0.000	0.364	Female	24
**19**	0.000	3.201	394.879	0.000	0.235	Female	30
**20**	0.000	10.633	721.689	0.000	0.372	Male	68
**24**	0.000	13.592	501.033	0.000	0.500	Male	45
**25**	10.323	4.950	337.031	0.000	0.429	Female	25
**27**	25.244	11.171	437.221	0.000	0.271	Female	25
**28**	0.000	5.151	438.414	0.000	0.262	Female	34
**30**	11.786	5.017	353.133	0.000	0.346	Female	27
**33**	0.000	5.026	573.241	0.000	0.454	Male	40
**34**	0.000	1.719	531.221	0.000	0.307	Female	24
**38**	0.000	3.587	869.350	0.000	0.245	Female	26
**39**	8.157	3.548	888.569	0.000	0.280	Female	48
**41**	4.650	5.551	611.978	0.000	0.571	Female	25
**43**	0.000	8.255	737.381	0.000	0.417	Female	24
**44**	0.000	8.877	947.723	0.000	0.394	Female	25
**46**	0.000	6.757	1,483.200	0.000	0.351	Male	43
**47**	0.000	8.138	704.553	0.000	0.330	Female	29
**48**	0.000	8.955	826.017	0.000	0.635	Female	28
**51**	3.375	7.360	741.732	0.000	0.153	Male	28
**52**	0.000	8.177	857.532	0.000	0.209	Female	35
**54**	0.000	4.929	1,234.749	12.793	0.173	Female	23
**55**	0.000	6.504	1,814.282	0.000	0.130	Male	32
**56**	0.000	7.127	855.562	0.000	0.234	Female	29
**60**	2.418	5.882	867.380	0.000	0.212	Female	26
**61**	0.000	8.994	833.239	0.000	0.184	Female	28
**63**	21.864	9.675	1,582.157	0.000	0.199	Male	37
**64**	0.000	3.606	1,368.139	0.000	0.240	Female	26

Table S1a (continued)

Number	AFP (ng/mL)	AFU (mU/mL)	GGT2 (mU/mL)	GPC3 (ng/mL)	HGF (ng/mL)	Gender	Age
**68**	0.000	4.326	824.047	0.000	0.229	Female	41
**71**	0.000	4.442	711.119	0.000	0.210	Female	30
**72**	0.825	5.609	827.330	0.000	0.193	Female	27
**73**	13.275	8.540	548.900	0.558	0.211	Male	26
**78**	19.292	2.717	530.420	0.000	0.222	Female	29
**80**	6.829	0.000	290.177	0.000	0.232	Female	31
**81**	24.449	9.319	397.664	0.156	0.220	Male	48
**83**	12.416	15.598	513.448	1.906	0.368	Male	54
**84**	3.391	4.349	407.093	0.558	0.172	Female	24
**85**	4.680	13.466	350.144	0.582	0.383	Female	33
**86**	39.062	16.186	372.018	0.000	0.316	Male	42
**87**	10.267	7.614	263.400	0.000	0.275	Female	29
**88**	20.581	4.143	443.299	1.078	0.164	Male	33
**92**	2.961	9.510	579.072	0.440	0.191	Female	25
**93**	13.705	6.026	434.248	0.000	0.155	Female	33
**95**	16.284	15.406	642.433	0.535	0.270	Male	32
**96**	30.466	6.746	568.889	0.000	0.186	Female	28
**98**	8.978	2.908	457.631	0.000	0.598	Female	26
**101**	4.250	3.879	235.491	0.000	0.412	Female	48
**103**	18.862	4.746	543.997	0.000	0.477	Male	26
**105**	8.548	1.100	491.197	0.000	0.384	Female	69
**106**	12.846	4.820	422.556	0.000	0.405	Male	83
**111**	4.250	2.776	375.790	0.416	0.416	Female	58
**112**	11.127	2.306	257.743	2.426	0.472	Female	63
**116**	0.000	3.031	1,194.775	0.000	0.414	Female	56
**118**	11.324	12.467	502.778	0.000	0.538	Male	68
**119**	0.000	3.672	415.533	0.000	0.357	Male	51
**121**	0.000	2.454	681.926	0.000	0.594	Female	66
**122**	0.000	1.589	601.881	0.000	0.441	Female	56
**124**	0.000	3.367	831.427	0.000	0.471	Female	64
**125**	3.074	3.928	760.276	0.000	0.379	Male	64
**128**	16.365	2.021	1,183.854	0.000	0.519	Female	59
**129**	0.000	3.095	567.576	0.000	0.741	Male	62
**131**	18.657	2.822	615.010	0.000	0.371	Female	65
**132**	0.000	4.697	738.253	0.133	0.450	Female	57

Table S1a (continued)

Number	AFP (ng/mL)	AFU (mU/mL)	GGT2 (mU/mL)	GPC3 (ng/mL)	HGF (ng/mL)	Gender	Age
**135**	1.241	3.127	456.614	0.000	0.386	Female	42
**138**	0.000	5.081	742.912	0.000	0.565	Female	63
**141**	0.000	2.614	744.183	0.000	0.385	Female	59
**142**	0.000	4.489	895.802	0.000	0.496	Female	58
**145**	0.000	5.081	902.313	0.000	0.167	Male	75
**146**	0.000	0.515	842.015	0.000	0.162	Female	62
**147**	20.032	3.287	602.728	0.000	0.236	Male	63
**151**	5.824	2.117	534.965	0.000	0.119	Female	25
**155**	0.000	1.486	412.283	0.000	0.222	Female	68
**158**	0.000	1.612	361.943	0.000	0.105	Male	19
**159**	0.000	1.517	331.942	0.000	0.137	Female	59
**161**	0.000	1.722	417.368	0.000	0.171	Female	63
**162**	0.000	5.505	490.590	0.000	0.179	Male	72
**163**	0.000	4.307	643.645	0.000	0.206	Female	73
**164**	0.000	5.378	621.780	0.000	0.178	Female	51
**165**	0.000	2.825	497.709	0.000	0.172	Female	61
**166**	0.000	6.387	460.589	0.000	0.188	Female	63
**170**	0.000	2.132	327.366	0.000	0.203	Male	67
**173**	0.000	5.410	440.758	0.000	0.169	Male	79
**174**	0.000	5.142	360.926	0.000	0.227	Female	69
**182**	0.000	0.000	250.075	0.000	0.149	Male	79
**183**	0.000	4.795	178.887	0.000	0.159	Female	61
**184**	0.000	3.172	329.399	0.421	0.166	Male	62
**185**	0.000	0.000	485.505	0.000	0.151	Female	58
**186**	0.000	5.898	345.671	0.000	0.192	Female	65
**191**	0.000	1.990	276.517	0.356	0.259	Female	69
**193**	0.000	7.058	733.206	0.000	0.511	Female	60
**196**	0.000	5.215	1,042.845	0.000	0.763	Female	70
**200**	5.258	9.515	458.420	0.000	0.650	Female	60
**201**	0.000	6.414	703.338	0.000	0.681	Male	68
**202**	0.000	5.858	624.828	0.000	0.581	Male	72
**203**	0.000	9.691	641.042	0.000	0.654	Female	77
**207**	0.000	6.999	6,310.173	0.184	0.489	Female	64
**209**	0.000	5.420	984.543	0.000	0.853	Female	61
**212**	0.000	6.034	910.708	0.000	0.506	Male	75
**214**	0.000	9.164	767.341	0.000	0.534	Male	85

Table S1a (continued)

Number	AFP (ng/mL)	AFU (mU/mL)	GGT2 (mU/mL)	GPC3 (ng/mL)	HGF (ng/mL)	Gender	Age
**216**	0.000	9.077	940.576	0.000	0.421	Male	74
**217**	31.529	4.366	2,609.853	0.000	0.782	Female	68
**218**	3.798	4.922	709.312	0.000	0.591	Female	64
**220**	0.000	3.284	633.361	0.000	0.458	Female	64
**221**	0.000	5.039	699.071	0.000	0.451	Female	50
**222**	0.000	5.478	839.024	0.000	0.471	Female	76
**223**	0.000	10.686	840.731	0.000	0.484	Male	40
**224**	7.204	6.882	751.980	0.000	0.433	Male	71
**225**	17.907	2.845	593.253	0.000	0.719	Male	70
**228**	9.150	8.608	752.834	0.000	0.601	Female	67
**229**	0.393	8.433	623.974	0.000	0.487	Male	68
**234**	0.000	8.158	373.547	0.000	0.499	Male	55
**236**	7.064	7.813	352.357	0.000	0.546	Female	57
**237**	0.000	11.062	563.081	0.000	0.787	Female	58
**238**	7.972	5.209	406.510	0.000	0.477	Female	63
**239**	0.707	8.366	703.171	0.000	0.671	Female	62
**240**	7.518	1.844	332.344	0.000	0.536	Male	66
**241**	12.059	10.785	591.334	0.000	0.757	Female	74
**243**	22.502	16.753	525.410	0.000	0.576	Male	75
**246**	4.340	12.744	697.285	0.000	0.791	Female	62
**248**	8.880	4.633	592.512	0.000	0.587	Female	65
**249**	9.789	12.905	467.726	0.000	0.936	Female	66
**250**	3.886	16.247	860.920	0.000	0.974	Male	55
**251**	12.513	7.767	531.296	0.000	0.688	Male	52
**257**	1.162	17.952	840.907	9.686	0.892	Male	50
**258**	1.162	15.325	651.373	15.446	0.951	Male	45
**259**	7.972	13.043	770.273	4.281	0.857	Male	59
**261**	0.000	14.080	767.919	5.973	0.711	Male	52
**263**	0.000	12.537	943.528	5.673	0.798	Male	58
**265**	26.588	17.952	544.245	14.354	1.176	Male	52
**266**	7.518	10.209	716.121	9.823	0.819	Male	47
**267**	7.064	14.380	777.447	29.521	0.707	Male	41
**270**	0.000	14.703	707.880	15.638	0.489	Male	50
**274**	26.315	9.557	394.531	0.000	0.855	Male	45
**275**	0.000	1.454	570.850	0.000	0.747	Female	41
**277**	5.034	0.968	431.558	0.000	0.527	Male	60

Table S1a (continued)

Number	AFP (ng/mL)	AFU (mU/mL)	GGT2 (mU/mL)	GPC3 (ng/mL)	HGF (ng/mL)	Gender	Age
**278**	20.313	3.443	406.874	40.221	0.689	Female	30
**279**	8.308	4.226	376.018	152.273	0.504	Female	30
**285**	7.217	5.939	1,741.841	58.902	0.617	Male	44
**287**	5.580	1.750	491.506	61.529	0.627	Male	28
**289**	6.671	1.708	577.902	55.979	0.221	Female	39
**291**	13.765	8.245	596.416	8.754	0.287	Female	44
**292**	8.308	7.209	469.467	32.677	0.376	Female	31
**293**	5.580	9.218	560.271	16.261	0.377	Male	26
**298**	31.772	4.204	515.309	42.915	0.443	Male	62
**302**	2.306	9.642	555.863	17.633	0.490	Male	56
**303**	14.856	8.076	372.492	2.366	0.457	Female	35
**304**	14.311	10.297	228.792	24.291	0.482	Male	51
**306**	4.489	7.272	632.561	27.777	0.465	Male	27
**308**	8.308	7.949	423.624	2.727	0.408	Male	30
**310**	0.000	12.455	507.375	6.372	0.451	Male	33
**312**	43.231	4.310	207.634	32.631	0.348	Male	54
**314**	0.000	2.864	352.573	25.491	0.302	Male	27
**317**	0.000	7.899	2,416.889	23.458	0.438	Male	31
**320**	0.000	7.899	674.299	23.967	0.650	Female	31
**323**	67.991	9.235	761.287	18.944	0.313	Female	62
**324**	0.000	7.248	773.440	10.672	0.305	Female	65
**326**	0.000	2.916	474.100	69.076	0.517	Female	30
**330**	0.000	2.881	797.745	14.659	0.307	Male	68
**331**	0.000	7.847	1,419.580	51.709	0.288	Male	40
**332**	0.000	6.974	827.807	22.966	0.459	Female	40
**334**	0.000	4.851	731.865	51.983	0.458	Female	33
**335**	0.000	3.875	764.485	36.921	0.471	Female	57
**337**	56.645	5.621	495.847	23.091	0.343	Female	70
**339**	0.000	6.152	702.602	23.722	0.319	Female	62
**340**	0.000	5.981	511.198	53.962	0.379	Female	43
**341**	0.000	3.977	564.286	1.438	0.258	Female	38
**342**	0.000	12.968	739.540	169.094	0.338	Male	41
**343**	0.000	3.412	685.173	49.386	0.359	Male	38
**345**	0.000	7.625	473.461	24.516	0.314	Male	58

Table S1b The information of 347 healthy controls (validation group)

Number	AFP (ng/mL)	AFU (mU/mL)	GGT2 (mU/mL)	GPC3 (ng/mL)	HGF (ng/mL)	Gender	Age
**1**	0.000	2.209	322.718	0.000	0.358	Female	24
**3**	0.000	6.362	502.822	0.000	0.263	Female	27
**7**	10.323	11.305	423.504	0.000	0.302	Female	42
**10**	0.000	7.001	512.960	0.000	0.424	Female	27
**11**	0.000	9.304	442.588	0.000	0.457	Male	52
**12**	0.000	10.196	523.695	0.000	0.347	Female	30
**13**	0.000	9.019	337.627	0.984	0.261	Female	24
**15**	0.000	5.219	567.230	0.000	0.310	Female	28
**16**	0.000	4.058	552.917	0.000	0.242	Female	26
**17**	36.947	2.579	279.779	0.000	0.307	Female	31
**21**	7.105	15.711	738.984	0.000	0.472	Male	54
**22**	12.956	3.100	246.383	0.000	0.236	Female	28
**23**	0.000	7.690	569.019	0.285	0.404	Female	55
**26**	12.371	5.958	384.144	0.000	0.314	Male	57
**29**	0.000	5.605	224.913	0.000	0.258	Female	31
**31**	55.964	10.212	383.548	0.000	0.222	Female	31
**32**	12.078	4.428	319.736	0.000	0.296	Female	48
**35**	0.000	5.357	494.454	0.000	0.353	Female	23
**36**	0.000	7.379	837.835	0.000	0.341	Female	24
**37**	35.572	8.099	622.483	0.000	0.382	Female	28
**40**	0.000	2.322	728.189	0.000	0.194	Female	26
**42**	0.000	5.357	1,445.981	0.000	0.459	Female	32
**45**	0.000	6.932	834.552	0.000	0.363	Female	35
**49**	34.616	4.870	803.037	0.000	0.189	Male	37
**50**	14.851	5.240	1,399.506	0.000	0.232	Male	28
**53**	0.000	9.519	726.689	0.000	0.256	Male	50
**57**	11.663	8.410	847.027	0.000	0.349	Male	45
**58**	0.000	3.840	742.634	0.000	0.166	Female	31
**59**	4.331	10.919	785.967	0.000	0.229	Female	27
**62**	0.000	5.901	1,380.162	0.000	0.228	Male	54
**65**	18.995	3.275	606.069	0.000	0.167	Female	35
**66**	9.750	2.206	795.158	0.000	0.211	Male	40
**67**	0.000	5.473	828.643	0.000	0.188	Male	43
**69**	0.000	6.115	908.743	0.000	0.223	Female	27

Table S1b (continued)

Number	AFP (ng/mL)	AFU (mU/mL)	GGT2 (mU/mL)	GPC3 (ng/mL)	HGF (ng/mL)	Gender	Age
**70**	0.000	11.950	857.532	0.000	0.271	Female	41
**74**	27.458	13.348	402.567	0.464	0.305	Female	43
**75**	4.250	4.937	451.973	0.000	0.117	Female	25
**76**	8.978	1.350	501.380	0.000	0.163	Female	42
**77**	19.292	1.247	411.619	0.000	0.129	Female	25
**79**	14.565	2.379	329.401	0.298	0.211	Male	31
**82**	5.110	8.996	618.295	0.133	0.200	Female	27
**89**	5.110	5.908	467.436	1.409	0.168	Female	56
**90**	0.000	7.863	364.852	0.298	0.221	Male	51
**91**	3.820	12.451	308.658	0.000	0.288	Female	30
**94**	4.680	19.200	454.613	0.606	0.389	Female	29
**97**	5.539	9.510	387.104	0.984	0.599	Male	27
**99**	5.969	11.745	387.858	0.653	0.746	Male	80
**100**	3.391	7.319	455.745	0.000	0.538	Female	41
**102**	5.539	3.291	516.088	0.000	0.479	Male	67
**104**	22.301	2.320	387.481	0.000	0.406	Female	62
**107**	6.829	6.820	469.322	0.000	0.501	Female	69
**108**	9.407	2.453	535.323	0.000	0.441	Female	77
**109**	16.284	2.350	597.929	0.000	0.413	Male	77
**110**	11.127	0.615	424.442	0.000	0.440	Male	63
**113**	0.000	2.406	407.063	0.000	0.471	Female	47
**114**	0.000	4.425	692.090	0.000	0.575	Female	68
**115**	0.000	2.854	586.211	0.000	0.371	Female	58
**117**	0.000	5.514	565.458	0.000	0.429	Female	75
**120**	7.657	1.525	509.977	0.000	0.435	Female	70
**123**	0.000	3.415	526.918	0.000	0.488	Female	80
**126**	3.532	5.049	533.694	0.000	0.498	Female	55
**127**	3.532	4.681	598.493	0.000	0.379	Male	63
**130**	0.000	1.044	731.901	0.000	0.449	Female	64
**133**	0.000	3.736	690.819	0.000	0.376	Female	59
**134**	0.000	7.292	529.459	0.000	0.384	Female	74
**136**	36.990	4.761	483.719	0.026	0.386	Male	43
**137**	3.991	4.809	745.030	0.000	0.626	Female	84
**139**	0.000	6.027	621.363	0.000	0.456	Female	63
**140**	0.000	3.015	576.470	0.000	0.487	Female	71

Table S1b (continued)

Number	AFP (ng/mL)	AFU (mU/mL)	GGT2 (mU/mL)	GPC3 (ng/mL)	HGF (ng/mL)	Gender	Age
**143**	0.000	5.658	537.930	0.000	0.462	Female	58
**144**	0.000	2.983	644.656	0.000	0.522	Female	80
**148**	0.000	0.000	566.305	0.000	0.171	Female	63
**149**	1.699	4.232	3,051.854	0.000	0.332	Female	75
**150**	0.000	2.630	472.284	0.000	0.203	Male	68
**152**	24.157	2.390	582.822	0.000	0.316	Female	62
**153**	8.780	1.722	442.792	0.000	0.153	Female	58
**154**	0.000	1.439	309.568	0.000	0.172	Male	48
**156**	47.907	5.962	457.030	0.726	0.178	Female	63
**157**	0.000	2.037	456.521	0.639	0.159	Female	61
**160**	0.000	2.274	486.014	0.000	0.170	Female	69
**167**	0.000	10.280	643.137	0.000	0.192	Female	51
**168**	0.000	12.108	516.523	0.465	0.208	Male	58
**169**	0.000	4.055	626.356	0.000	0.276	Male	80
**171**	0.000	2.967	467.200	0.000	0.150	Male	73
**172**	0.000	4.086	736.698	0.182	0.135	Female	59
**175**	0.000	19.168	222.109	0.508	0.366	Male	77
**176**	0.000	3.078	316.179	0.030	0.147	Male	56
**177**	33.392	3.802	482.454	0.008	0.222	Male	66
**178**	0.000	2.211	377.197	0.000	0.300	Female	60
**179**	0.000	5.126	529.235	0.008	0.237	Male	78
**180**	0.000	3.566	362.451	0.000	0.178	Female	74
**181**	0.000	2.164	629.407	0.000	0.154	Female	77
**187**	0.000	0.588	359.400	0.000	0.172	Female	52
**188**	0.000	0.000	408.724	0.000	0.157	Female	57
**189**	0.000	4.196	474.827	0.000	0.180	Female	61
**190**	0.000	3.755	319.230	0.000	0.186	Female	60
**192**	1.838	4.228	296.856	0.247	0.192	Female	56
**194**	0.000	7.906	612.027	0.000	0.782	Male	53
**195**	0.000	9.106	657.256	0.000	0.761	Male	46
**197**	0.000	9.984	637.628	0.499	0.688	Female	82
**198**	11.582	1.616	658.963	0.000	0.477	Female	62
**199**	0.000	6.005	616.294	0.000	0.595	Female	56
**204**	0.000	6.209	516.449	0.000	0.499	Female	63
**205**	2.825	6.092	806.596	0.000	0.452	Male	68

Table S1b (continued)

Number	AFP (ng/mL)	AFU (mU/mL)	GGT2 (mU/mL)	GPC3 (ng/mL)	HGF (ng/mL)	Gender	Age
**206**	5.744	9.106	760.514	0.355	0.489	Female	61
**208**	0.000	3.986	577.892	0.000	0.458	Female	73
**210**	0.393	5.215	1,589.362	0.000	0.589	Female	59
**211**	2.825	9.925	850.971	0.000	0.534	Female	67
**213**	0.000	8.550	842.438	0.000	0.506	Female	66
**215**	3.312	6.970	829.637	0.000	0.416	Female	61
**219**	7.204	7.204	677.737	0.000	0.494	Male	62
**226**	1.366	7.848	733.206	0.000	0.638	Female	56
**227**	58.774	2.845	749.420	0.000	0.484	Female	63
**230**	12.069	9.749	810.010	0.000	0.518	Female	61
**231**	11.096	1.616	891.080	0.000	0.451	Male	66
**232**	2.339	3.723	651.282	0.000	0.471	Female	73
**233**	17.053	8.757	520.701	0.000	0.959	Female	55
**235**	4.340	6.822	460.662	0.000	0.749	Female	57
**242**	5.248	11.799	1,428.173	0.000	0.870	Male	49
**244**	5.248	7.467	690.222	0.000	0.515	Male	70
**245**	0.000	8.435	530.119	0.000	0.586	Female	61
**247**	7.972	12.583	620.765	0.377	0.790	Female	70
**252**	1.162	24.018	714.943	0.000	0.763	Male	52
**253**	10.697	16.477	458.308	0.000	0.854	Male	53
**254**	0.000	12.191	606.638	0.000	0.841	Female	74
**255**	5.702	13.182	790.286	9.195	0.801	Male	58
**256**	7.972	10.324	786.754	0.000	0.592	Male	51
**260**	24.772	14.587	692.576	2.779	0.604	Male	51
**262**	0.000	9.449	664.323	4.881	0.532	Male	45
**264**	0.000	3.411	344.117	1.551	0.451	Male	51
**268**	12.967	20.486	742.020	52.528	0.729	Male	51
**269**	0.000	13.251	701.994	14.873	0.566	Male	52
**271**	0.000	8.343	586.626	23.363	0.688	Male	43
**272**	0.000	12.168	672.563	68.580	0.716	Male	46
**273**	0.000	5.664	361.031	23.083	0.995	Male	46
**276**	2.306	7.991	470.348	1.464	0.663	Male	46
**280**	15.948	8.457	351.333	7.924	0.548	Male	71
**281**	0.123	6.532	517.073	15.106	0.938	Female	20
**282**	7.763	2.893	506.493	37.261	0.793	Female	23

Table S1b (continued)

Number	AFP (ng/mL)	AFU (mU/mL)	GGT2 (mU/mL)	GPC3 (ng/mL)	HGF (ng/mL)	Gender	Age
**283**	1.215	3.189	305.491	54.828	0.711	Female	26
**284**	12.674	5.030	805.353	52.333	0.509	Male	40
**286**	5.034	2.639	592.008	267.161	0.627	Female	28
**288**	6.126	3.041	153.857	39.622	0.653	Female	22
**290**	10.491	34.149	1,097.318	335.264	0.897	Male	27
**294**	18.676	10.234	851.196	9.909	0.467	Male	30
**295**	23.041	8.901	354.860	13.446	0.411	Male	34
**296**	21.950	2.173	223.503	4.207	0.235	Male	33
**297**	7.217	1.095	606.995	10.054	0.218	Male	42
**299**	25.769	8.266	536.468	16.586	0.338	Male	25
**300**	14.856	7.314	356.623	22.649	0.373	Female	37
**301**	9.945	5.156	420.979	13.410	0.252	Male	33
**305**	0.000	8.922	500.322	15.539	0.675	Male	62
**307**	6.671	14.782	366.320	23.263	0.418	Male	41
**309**	0.000	8.372	466.822	15.828	0.489	Male	28
**311**	14.856	9.070	199.700	21.603	0.699	Male	51
**313**	0.000	11.170	576.439	44.420	0.391	Male	47
**315**	0.000	2.590	865.776	38.233	0.395	Male	30
**316**	0.000	4.868	898.607	12.528	0.318	Female	35
**318**	0.000	5.621	742.099	16.973	0.415	Female	66
**319**	0.000	13.995	532.305	40.362	0.379	Male	60
**321**	0.000	2.642	570.682	1.094	0.475	Female	47
**322**	0.000	5.056	513.756	23.366	0.540	Female	41
**325**	57.185	0.000	878.336	4.806	0.344	Female	40
**327**	0.000	3.121	634.004	0.063	0.578	Female	46
**328**	0.000	1.871	680.056	7.487	0.369	Female	31
**329**	0.000	6.032	738.901	22.679	0.291	Male	31
**333**	0.000	5.227	576.439	106.452	0.337	Female	42
**336**	0.000	12.933	641.040	176.700	0.283	Female	33
**338**	0.000	1.152	593.708	66.447	0.271	Female	47
**344**	0.000	17.249	843.158	110.541	0.384	Male	35
**346**	0.000	2.196	478.578	12.528	0.209	Male	61
**347**	0.000	8.156	728.027	20.090	0.323	Male	69

Table S2a The information of 54 NBNC-HCC patients (test group)

Number	AFP (ng/mL)	AFU (mU/mL)	GGT2 (mU/mL)	GPC3 (ng/mL)	HGF (ng/mL)	Gender	Age
**9**	1,895.072	479.602	14,431.953	38.260	4.291	Female	68
**620**	103.799	8.725	711.468	4.045	0.238	Female	49
**545**	88.895	9.277	7,278.588	3.062	0.508	Female	65
**395**	1,303.483	174.560	12,793.993	16.906	2.034	Female	77
**590**	1,245.818	36.586	4,023.670	30.478	0.311	Male	54
**586**	427.605	16.869	1,507.894	51.215	0.570	Male	60
**574**	435.096	17.330	9,471.676	13.364	0.346	Male	73
**543**	893.821	32.685	1,643.010	26.444	1.053	Male	69
**132**	55.332	32.535	702.669	22.067	0.525	Male	62
**94**	0.000	33.453	291.770	5.691	1.195	Male	40
**72**	5,887.511	49.575	921.998	21.104	0.733	Male	56
**45**	9,630.275	192.102	5,128.398	5.418	1.461	Male	61
**19**	0.000	253.009	7,026.921	31.044	1.578	Male	72
**18**	0.000	43.676	762.983	6.796	1.029	Male	49
**65**	3,208.245	29.461	1,296.319	0.720	0.848	Male	57
**589**	9.957	7.497	724.291	10.818	0.223	Male	47
**473**	11.526	78.777	1,401.820	6.096	0.392	Male	49
**459**	367.386	30.063	9,080.726	8.021	0.441	Male	79
**367**	14.006	26.425	538.084	1.084	0.371	Male	66
**195**	5,966.582	384.668	13,014.503	15.682	1.325	Male	73
**193**	0.000	202.639	634.542	7.732	1.137	Male	64
**157**	7.859	14.117	671.018	22.448	0.616	Male	67
**129**	2.584	21.124	842.257	19.302	0.703	Male	52
**115**	0.000	21.124	711.596	1.007	0.475	Male	57
**8**	0.000	26.625	638.297	18.225	0.936	Male	61
**598**	6,626.332	28.049	15,256.585	18.983	0.299	Male	58
**87**	7,673.842	71.793	1,768.286	12.708	1.100	Male	58

Table S2b The information of 54 NBNC-HCC patients (validation group)

Number	AFP (ng/mL)	AFU (mU/mL)	GGT2 (mU/mL)	GPC3 (ng/mL)	HGF (ng/mL)	Gender	Age
**636**	7,357.865	8.035	599.087	19.989	0.218	Female	62
**628**	0.000	8.280	451.785	1.467	0.208	Female	39
**542**	3.727	17.651	687.167	5.365	0.398	Female	62
**393**	182.199	18.098	977.582	5.289	0.474	Female	52
**294**	0.000	487.276	5,452.020	10.940	2.870	Female	55
**231**	7.795	321.586	625.718	129.202	1.794	Female	76
**229**	0.000	80.686	584.808	17.504	1.036	Female	72
**80**	44.989	47.079	1,860.445	12.291	0.631	Female	50
**646**	64.684	10.706	729.246	2.868	0.236	Male	40
**616**	0.000	76.231	2,331.890	236.046	1.306	Male	70
**603**	0.000	12.614	811.063	6.311	0.321	Male	21
**572**	3,304.545	8.781	6,867.373	20.216	0.297	Male	65
**556**	0.000	17.386	906.014	4.151	0.374	Male	77
**524**	892.291	14.432	8,949.422	139.367	0.605	Male	63
**523**	5,804.144	66.970	2,510.479	16.133	1.118	Male	60
**486**	33.048	19.702	618.687	2.651	0.538	Male	47
**463**	0.000	13.065	636.543	55.485	0.302	Male	54
**422**	65.080	18.596	765.263	6.572	0.350	Male	66
**373**	735.086	59.020	805.772	3.048	0.519	Male	63
**203**	375.112	625.725	5,492.826	13.993	0.983	Male	72
**185**	0.000	169.128	1,489.102	13.794	0.749	Male	52
**138**	0.000	69.744	784.637	18.358	1.418	Male	59
**73**	72.010	21.360	248.074	1.597	0.479	Male	66
**57**	2.921	32.959	294.995	0.000	0.920	Male	58
**23**	9,294.590	36.968	743.033	58.214	0.705	Male	70
**11**	6,119.195	76.334	2,671.297	37.285	1.083	Male	62
**5**	10,817.572	121.048	1,639.530	10.541	1.482	Male	77

Table S3a The information of 154 hepatitis patients (test group)

Number	AFP (ng/mL)	AFU (mU/mL)	GGT2 (mU/mL)	GPC3 (ng/mL)	HGF (ng/mL)	Gender	Age
**4**	0.000	33.712	2,296.099	6.708	0.930	Female	56
**91**	0.000	15.270	771.755	47.542	0.826	Female	62
**148**	0.000	13.256	370.937	0.000	0.802	Female	75
**80**	0.000	19.843	258.479	0.288	0.722	Female	55
**149**	0.000	8.476	436.495	14.507	0.612	Female	73
**6**	0.000	3.930	360.443	0.193	0.601	Female	81
**55**	0.000	20.249	503.238	24.519	0.591	Female	27
**30**	19.281	4.121	2,852.941	1.052	0.588	Female	55
**28**	0.000	10.349	2,130.785	8.013	0.548	Female	62
**43**	448.103	18.947	330.408	16.005	0.510	Female	63
**10**	0.000	0.834	4,419.132	14.608	0.506	Female	58
**111**	0.000	11.457	192.518	2.551	0.476	Female	30
**12**	1.934	6.681	630.131	10.183	0.451	Female	60
**64**	0.000	3.870	1,340.159	21.521	0.441	Female	58
**74**	0.000	5.246	812.586	0.000	0.415	Female	33
**152**	0.000	5.107	79.650	124.536	0.406	Female	49
**103**	0.000	14.961	575.769	249.865	0.387	Female	88
**21**	0.000	0.624	924.221	0.537	0.374	Female	34
**75**	0.000	6.984	475.170	0.354	0.365	Female	63
**145**	0.000	9.730	1,012.364	16.331	0.360	Female	67
**31**	8.892	7.483	525.635	15.489	0.352	Female	66
**41**	0.000	0.000	528.137	0.000	0.342	Female	66
**127**	0.000	12.848	266.911	11.830	0.331	Female	53
**63**	0.000	8.924	593.415	6.157	0.308	Female	55
**46**	0.000	3.261	525.635	4.189	0.295	Female	57
**132**	15.551	5.629	238.331	8.599	0.275	Female	56
**51**	0.000	4.254	457.254	0.000	0.274	Female	62
**123**	0.000	12.719	260.241	12.164	0.270	Female	82
**113**	0.000	16.945	295.642	6.144	0.267	Female	36
**118**	0.000	7.721	329.504	43.163	0.254	Female	36
**102**	0.000	14.652	426.471	15.304	0.246	Female	71
**146**	0.000	5.420	296.439	33.829	0.211	Female	68
**88**	0.000	2.593	396.200	0.029	0.193	Female	54
**47**	0.000	6.961	620.288	0.000	0.188	Female	58
**119**	0.000	3.160	144.804	2.979	0.159	Female	43
**84**	0.000	0.509	5,386.430	0.000	0.070	Female	50

Table S3a (continued)

Number	AFP (ng/mL)	AFU (mU/mL)	GGT2 (mU/mL)	GPC3 (ng/mL)	HGF (ng/mL)	Gender	Age
**8**	0.000	10.598	712.101	28.676	0.988	Male	22
**20**	9.691	17.820	661.417	0.665	0.987	Male	63
**7**	0.000	12.145	2,690.393	89.557	0.966	Male	80
**83**	0.000	13.052	742.714	0.000	0.865	Male	40
**33**	0.000	12.413	2,794.616	1.675	0.782	Male	38
**106**	0.000	26.504	657.857	58.107	0.739	Male	62
**141**	0.000	18.559	445.435	29.456	0.703	Male	60
**77**	0.000	16.730	1,547.571	81.292	0.665	Male	37
**16**	11.689	12.566	3,408.180	91.712	0.568	Male	66
**99**	0.000	25.978	674.788	14.424	0.537	Male	43
**67**	0.000	10.413	2,306.657	9.036	0.523	Male	33
**62**	0.000	30.063	426.199	0.442	0.519	Male	44
**85**	0.000	12.353	690.758	13.191	0.515	Male	53
**128**	0.232	9.234	569.101	2.665	0.492	Male	77
**44**	49.250	7.713	4,151.063	1.632	0.447	Male	43
**130**	0.000	12.891	1,631.498	63.595	0.428	Male	40
**140**	0.000	19.264	814.944	12.041	0.426	Male	30
**32**	0.000	13.846	3,416.174	8.550	0.418	Male	49
**122**	0.000	15.296	864.618	14.781	0.417	Male	69
**14**	0.901	3.605	830.363	10.612	0.404	Male	29
**155**	20.338	6.805	228.646	24.076	0.396	Male	54
**95**	0.000	7.206	328.477	3.289	0.389	Male	56
**36**	0.000	8.133	2,345.740	0.601	0.369	Male	27
**109**	0.000	16.172	392.096	8.928	0.348	Male	64
**164**	5.328	0.000	768.046	29.778	0.343	Male	63
**169**	0.000	0.124	1,351.050	19.362	0.337	Male	49
**79**	0.000	18.895	2,140.239	9.806	0.330	Male	25
**150**	0.000	13.256	877.523	199.148	0.322	Male	44
**86**	0.000	4.863	332.440	0.794	0.317	Male	55
**171**	401.717	7.307	2,615.363	39.726	0.314	Male	41
**131**	0.000	6.387	510.993	24.850	0.302	Male	62
**161**	0.000	2.981	1,060.837	23.174	0.297	Male	49
**69**	0.000	6.036	1,064.152	17.147	0.283	Male	25
**135**	0.000	3.488	328.473	0.918	0.277	Male	57
**129**	0.000	6.700	420.851	0.000	0.271	Male	20

Table S3a (continued)

Number	AFP (ng/mL)	AFU (mU/mL)	GGT2 (mU/mL)	GPC3 (ng/mL)	HGF (ng/mL)	Gender	Age
**170**	0.000	4.629	2,065.907	10.217	0.250	Male	52
**66**	0.000	4.096	1,257.576	4.706	0.243	Male	29
**37**	0.000	3.968	1,944.007	3.437	0.240	Male	30
**163**	0.000	2.582	820.856	21.575	0.237	Male	66
**138**	0.000	4.349	486.409	0.000	0.222	Male	61
**162**	0.000	2.417	850.125	7.125	0.210	Male	42
**120**	0.000	6.046	266.911	0.243	0.202	Male	31
**42**	0.000	1.255	4,137.875	0.687	0.202	Male	61
**53**	13.169	2.765	5,400.203	0.000	0.156	Male	30
**90**	63.563	17.795	1,243.409	21.300	0.513	Male	40
**100**	42.340	84.894	4,935.180	5.597	0.876	Male	46

Table S3b The information of 154 hepatitis patients (validation group)

Number	AFP (ng/mL)	AFU (mU/mL)	GGT2 (mU/mL)	GPC3 (ng/mL)	HGF (ng/mL)	Gender	Age
**98**	474.436	25.202	486.498	23.513	0.600	Female	32
**48**	27.915	7.660	3,236.716	6.245	0.437	Female	46
**15**	25.275	7.866	1,239.505	11.859	0.843	Female	26
**78**	9.072	10.458	686.577	11.894	0.731	Female	60
**17**	2.499	4.445	785.310	7.927	0.421	Female	48
**73**	0.000	7.277	3,029.426	3.607	0.987	Female	27
**19**	0.000	22.024	790.942	0.000	0.970	Female	43
**2**	0.000	8.036	722.910	34.394	0.791	Female	64
**26**	0.000	23.017	5,820.360	5.972	0.763	Female	29
**49**	0.000	13.052	620.886	12.443	0.715	Female	38
**114**	0.000	24.031	950.297	15.399	0.611	Female	66
**96**	0.000	12.307	543.960	83.425	0.572	Female	35
**13**	0.000	6.089	561.301	7.261	0.549	Female	30
**112**	0.000	11.869	285.894	0.434	0.485	Female	27
**168**	0.000	10.081	830.400	14.776	0.464	Female	46
**156**	0.000	8.738	843.999	31.749	0.388	Female	47
**158**	0.000	2.129	1,126.420	3.099	0.371	Female	57
**157**	0.000	13.283	1,495.704	0.071	0.366	Female	81
**68**	0.000	7.954	948.309	5.871	0.350	Female	57
**94**	0.000	9.937	343.869	9.070	0.333	Female	27
**154**	0.000	5.499	382.112	0.000	0.315	Female	32
**151**	0.000	11.167	596.666	7.597	0.277	Female	47
**167**	0.000	2.802	902.299	4.379	0.274	Female	67
**52**	0.000	1.140	554.597	0.000	0.253	Female	20
**133**	0.000	4.167	373.172	49.701	0.250	Female	30
**124**	0.000	6.278	385.939	24.917	0.238	Female	55
**72**	0.000	1.637	607.747	0.000	0.221	Female	25
**45**	0.000	1.350	1,093.725	2.406	0.204	Female	44
**153**	0.000	10.279	408.186	0.000	0.190	Female	68
**101**	219.763	17.589	518.307	100.118	0.535	Male	28
**136**	17.466	14.745	2,471.872	59.010	0.397	Male	49
**11**	13.288	0.000	718.417	14.403	0.312	Male	30
**38**	8.493	6.413	787.188	21.891	0.346	Male	17
**142**	5.019	7.327	471.509	27.539	0.321	Male	57
**87**	4.976	0.000	388.576	0.000	0.180	Male	28
**39**	0.101	3.968	2,220.762	3.910	0.198	Male	36

Table S3b (continued)

Number	AFP (ng/mL)	AFU (mU/mL)	GGT2 (mU/mL)	GPC3 (ng/mL)	HGF (ng/mL)	Gender	Age
**104**	0.000	16.610	222.275	294.007	0.963	Male	20
**22**	0.000	6.394	825.357	0.000	0.632	Male	28
**126**	0.000	20.295	288.459	71.460	0.542	Male	71
**60**	0.000	12.646	3,580.859	18.686	0.519	Male	42
**125**	0.000	11.818	2,476.641	0.291	0.518	Male	45
**58**	0.000	2.898	15,138.761	91.864	0.510	Male	38
**82**	0.000	6.374	722.409	236.751	0.502	Male	63
**59**	0.000	14.947	1,801.335	0.000	0.449	Male	44
**35**	0.000	7.694	851.637	1.353	0.443	Male	40
**166**	0.000	5.357	1,247.496	1.260	0.427	Male	51
**108**	0.000	17.331	502.402	22.689	0.417	Male	29
**144**	0.000	10.775	315.064	102.886	0.416	Male	51
**5**	0.000	4.217	502.243	64.780	0.414	Male	24
**137**	0.000	16.939	622.740	38.144	0.404	Male	43
**50**	0.000	7.164	449.490	0.000	0.390	Male	48
**34**	0.000	20.075	17,961.375	17.229	0.382	Male	43
**81**	0.000	5.630	423.811	0.000	0.361	Male	40
**143**	0.000	18.115	382.857	222.017	0.355	Male	88
**40**	0.000	7.292	728.369	2.320	0.342	Male	57
**56**	0.000	29.960	810.948	0.000	0.339	Male	50
**70**	0.000	9.059	924.720	18.642	0.331	Male	52
**93**	0.000	8.700	236.128	7.833	0.328	Male	66
**165**	0.000	0.000	876.848	10.644	0.305	Male	42
**147**	0.000	10.331	394.031	20.402	0.300	Male	42
**27**	0.000	15.852	904.824	1.740	0.297	Male	59
**139**	0.000	8.529	309.849	0.000	0.289	Male	28
**76**	0.000	5.224	776.754	0.926	0.283	Male	25
**116**	0.000	14.446	491.115	15.447	0.272	Male	51
**134**	0.000	6.230	1,461.243	31.083	0.271	Male	23
**89**	0.000	4.964	308.981	2.051	0.270	Male	54
**160**	0.000	1.566	754.685	22.801	0.264	Male	60
**110**	0.000	22.356	597.317	1.671	0.246	Male	32
**29**	0.000	2.688	1,038.700	1.396	0.234	Male	24
**121**	0.000	4.912	495.733	0.077	0.211	Male	30
**92**	0.000	13.209	635.283	18.469	0.128	Male	62
**54**	46.758	18.805	611.928	6.047	0.812	Male	42

Table S4a The information of 122 liver cirrhosis patients (test group)

Number	AFP (ng/mL)	AFU (mU/mL)	GGT2 (mU/mL)	GPC3 (ng/mL)	HGF (ng/mL)	Gender	Age
**66**	0.000	25.480	750.370	7.247	0.770	Female	58
**132**	11.959	16.730	791.360	0.137	0.678	Female	66
**88**	40.886	17.747	756.035	63.879	0.674	Female	52
**64**	0.000	18.221	2,808.378	4.731	0.586	Female	60
**111**	0.000	20.965	806.361	151.712	0.584	Female	58
**50**	0.000	8.039	517.043	4.731	0.488	Female	68
**109**	0.000	5.481	1,767.259	113.406	0.470	Female	53
**130**	38.686	10.771	607.834	5.060	0.446	Female	58
**97**	0.000	9.132	371.566	66.681	0.439	Female	47
**34**	0.000	15.931	4,091.807	2.003	0.420	Female	57
**113**	23.837	5.865	799.924	14.529	0.402	Female	61
**12**	0.000	20.872	5,950.131	42.933	0.377	Female	56
**131**	29.390	9.690	2,284.608	2.372	0.374	Female	63
**93**	0.000	9.996	537.759	27.150	0.353	Female	52
**28**	0.000	12.159	1,389.649	0.000	0.320	Female	67
**39**	15.573	11.011	8,781.347	1.249	0.317	Female	58
**36**	0.000	8.862	687.157	0.000	0.309	Female	55
**15**	0.000	9.781	9,807.197	52.948	0.307	Female	34
**17**	0.000	11.154	3,213.889	37.136	0.276	Female	64
**134**	74.709	22.569	1,444.856	65.761	0.227	Female	44
**25**	11.067	17.448	6,662.214	33.222	0.730	Female	62
**61**	0.000	20.843	1,918.679	7.499	0.631	Female	61
**44**	0.000	11.341	3,559.411	0.000	0.537	Female	43
**3**	0.000	16.444	2,139.721	109.676	0.425	Female	69
**42**	0.000	21.064	3,182.935	8.757	0.386	Female	74
**2**	0.000	14.763	3,843.661	71.194	0.344	Female	55
**128**	19.319	16.054	10,257.127	0.089	0.330	Female	52
**136**	20.481	6.928	441.131	96.684	0.157	Female	56
**86**	0.000	14.928	634.315	148.098	0.736	Male	48
**147**	86.717	10.276	866.809	30.909	0.697	Male	51
**59**	0.000	8.972	746.686	0.094	0.566	Male	58
**125**	26.678	22.857	790.340	30.523	0.550	Male	71
**30**	18.953	13.942	3,593.142	1.788	0.537	Male	50
**96**	0.000	10.188	626.708	34.205	0.494	Male	61
**92**	0.000	12.398	3,653.862	1.139	0.485	Male	53
**168**	0.000	7.051	730.635	16.419	0.466	Male	57

Table S4a (continued)

Number	AFP (ng/mL)	AFU (mU/mL)	GGT2 (mU/mL)	GPC3 (ng/mL)	HGF (ng/mL)	Gender	Age
**107**	31.144	14.736	4,670.679	20.631	0.451	Male	63
**90**	0.000	23.095	503.818	22.036	0.446	Male	37
**77**	0.000	3.125	328.540	3.006	0.443	Male	55
**149**	37.524	4.242	661.872	5.298	0.415	Male	39
**20**	0.000	153.755	2,824.100	7.284	0.375	Male	81
**41**	0.000	6.149	536.078	17.563	0.327	Male	51
**116**	0.000	7.466	686.982	0.000	0.269	Male	60
**7**	0.000	15.296	1,336.447	13.823	0.195	Male	40
**114**	0.000	7.098	493.870	61.342	0.165	Male	63
**148**	58.828	27.544	5,168.250	86.835	1.565	Male	48
**68**	0.000	19.825	596.252	0.000	0.919	Male	36
**85**	0.000	38.537	13,256.484	55.564	0.873	Male	83
**115**	150.484	21.077	3,670.743	19.226	0.711	Male	53
**95**	0.000	16.882	8,772.287	115.475	0.608	Male	59
**45**	0.000	8.316	767.562	189.195	0.526	Male	51
**89**	45.757	12.622	4,459.584	33.976	0.518	Male	49
**84**	0.000	12.959	695.760	105.751	0.513	Male	64
**83**	5.977	19.188	2,134.075	7.724	0.484	Male	63
**35**	1.678	78.515	4,407.081	4.302	0.464	Male	58
**80**	0.000	11.038	4,075.448	2.790	0.456	Male	69
**119**	35.203	4.055	926.606	38.065	0.396	Male	52
**72**	0.000	4.385	537.306	2.287	0.382	Male	53
**102**	0.000	13.487	857.857	263.322	0.283	Male	64
**122**	13.896	12.602	1,884.033	0.588	0.259	Male	62
**156**	35.200	6.013	620.069	9.365	0.461	Male	47

Table S4b The information of 122 liver cirrhosis patients (validation group)

Number	AFP (ng/mL)	AFU (mU/mL)	GGT2 (mU/mL)	GPC3 (ng/mL)	HGF (ng/mL)	Gender	Age
**144**	84.393	14.929	612.932	53.989	0.389	Female	66
**31**	35.854	22.471	6,648.916	1.895	0.419	Female	56
**158**	20.093	8.444	843.869	12.719	0.349	Female	41
**129**	19.319	6.673	665.441	0.000	0.353	Female	57
**159**	14.670	19.687	401.877	4.870	0.375	Female	69
**38**	13.696	4.391	4,390.777	0.351	0.532	Female	56
**123**	12.346	10.261	694.499	23.946	0.314	Female	58
**1**	8.438	57.606	13,737.133	2.686	0.539	Female	62
**127**	6.923	12.152	556.345	1.397	0.276	Female	64
**10**	4.307	55.399	9,944.413	244.758	0.739	Female	67
**26**	4.307	14.332	12,638.712	2.758	0.503	Female	79
**106**	2.324	9.100	926.324	168.472	0.429	Female	58
**108**	1.512	21.174	10,330.802	41.274	0.634	Female	79
**33**	0.000	95.614	3,015.989	22.481	0.584	Female	64
**73**	0.000	12.878	732.563	2.898	0.488	Female	62
**57**	0.000	17.138	789.667	176.297	0.472	Female	56
**164**	0.000	7.203	910.142	4.170	0.454	Female	57
**5**	0.000	48.923	1,265.965	13.248	0.448	Female	80
**51**	0.000	10.887	848.613	115.078	0.440	Female	65
**58**	0.000	16.886	793.965	2.682	0.412	Female	68
**13**	0.000	11.544	5,664.016	13.284	0.278	Female	62
**69**	0.000	15.903	1,466.135	23.710	0.901	Female	77
**60**	0.000	53.580	1,309.998	15.658	0.842	Female	78
**37**	0.000	7.649	2,003.123	2.111	0.722	Female	60
**65**	0.000	6.754	2,124.246	1.927	0.521	Female	38
**54**	0.000	19.154	2,491.420	6.313	0.516	Female	76
**100**	0.000	7.786	1,080.498	11.193	0.509	Female	50
**43**	0.000	9.047	1,619.109	0.777	0.482	Female	56
**121**	7,087.474	45.786	2,327.570	4.442	0.551	Male	63
**56**	529.387	17.339	5,001.452	4.300	0.587	Male	55
**135**	155.277	11.762	791.870	57.486	0.246	Male	52
**126**	84.005	10.921	7,268.692	0.000	0.249	Male	65
**137**	63.476	15.769	694.499	55.374	0.733	Male	41
**120**	46.569	9.420	589.841	0.086	0.318	Male	48
**19**	33.225	15.193	4,705.558	54.897	0.676	Male	61
**32**	26.089	56.840	6,366.152	10.410	0.683	Male	59

Table S4b (continued)

Number	AFP (ng/mL)	AFU (mU/mL)	GGT2 (mU/mL)	GPC3 (ng/mL)	HGF (ng/mL)	Gender	Age
**22**	13.320	193.434	4,003.065	281.905	0.599	Male	71
**29**	9.565	13.409	2,483.094	0.351	0.327	Male	77
**11**	5.433	12.774	2,646.735	19.858	0.621	Male	49
**27**	4.682	7.485	701.973	0.000	0.304	Male	58
**105**	3.947	3.399	1,976.577	1.534	0.174	Male	71
**99**	1.918	9.820	725.605	0.000	0.384	Male	60
**162**	0.961	187.848	10,392.088	37.033	1.954	Male	53
**82**	0.700	18.724	825.087	125.973	1.050	Male	46
**87**	0.294	9.820	1,356.832	3.773	0.375	Male	31
**91**	0.000	14.336	533.078	324.506	0.581	Male	60
**46**	0.000	51.457	645.373	134.265	0.569	Male	59
**76**	0.000	2.797	366.609	14.867	0.491	Male	56
**75**	0.000	5.620	291.699	0.274	0.469	Male	24
**55**	0.000	17.339	739.932	130.167	0.446	Male	65
**101**	0.000	9.180	755.449	22.519	0.443	Male	63
**94**	0.000	12.062	845.568	37.708	0.422	Male	29
**79**	0.000	5.796	1,029.354	2.215	0.400	Male	39
**118**	0.000	11.646	612.078	4.563	0.326	Male	55
**71**	0.000	11.895	601.164	35.859	0.309	Male	28
**62**	0.000	35.971	574.147	7.714	0.397	Male	64
**110**	0.000	51.260	1,465.170	67.278	0.338	Male	51
**48**	0.000	7.737	824.666	1.424	0.265	Male	50
**98**	52.840	39.656	631.885	6.032	0.200	Male	60
**141**	49.919	13.022	1,056.713	3.704	0.135	Male	58
**103**	51.440	9.932	1,608.696	17.075	0.572	Male	53

Table S5a The information of 244 hepatitis HCC (test group)

Number	AFP (ng/mL)	AFU (mU/mL)	GGT2 (mU/mL)	GPC3 (ng/mL)	HGF (ng/mL)	Gender	Age
**553**	545.774	21.156	770.980	9.449	0.822	Female	61
**625**	0.000	34.861	2,334.766	7.940	0.764	Female	66
**215**	5,139.339	20.564	738.022	9.984	0.729	Female	61
**205**	0.000	252.200	282.390	13.529	0.695	Female	60
**641**	1,353.368	10.239	482.896	8.193	0.656	Female	60
**166**	0.000	15.244	341.524	7.952	0.621	Female	60
**549**	0.000	19.994	1,715.524	9.524	0.619	Female	62
**548**	11.712	12.761	1,783.750	2.914	0.605	Female	62
**219**	781.657	14.984	187.734	9.951	0.470	Female	59
**158**	57.593	18.964	2,396.014	20.125	0.446	Female	51
**405**	2,430.981	16.024	552.622	14.863	0.386	Female	35
**660**	12.794	2.033	2,245.969	27.692	0.363	Female	54
**624**	129.614	6.922	1,094.828	4.886	0.304	Female	67
**645**	4,673.718	9.103	434.008	3.709	0.286	Female	35
**189**	386.961	14.101	400.309	8.229	0.266	Female	60
**571**	5,963.545	48.031	12,111.739	70.245	1.571	Female	23
**271**	9,559.211	49.808	3,695.517	1.829	1.314	Female	51
**175**	0.000	52.577	526.249	44.585	1.120	Female	53
**256**	2,560.416	20.516	2,304.106	1.490	1.074	Female	57
**28**	71.590	18.400	673.209	10.364	0.974	Female	38
**51**	1,748.145	16.248	467.061	0.000	0.920	Female	52
**482**	1,151.405	18.790	3,324.837	10.040	0.829	Female	59
**183**	1,856.493	287.940	618.499	67.007	0.830	Female	56
**597**	0.000	13.276	1,648.955	9.989	0.521	Female	63
**528**	6,504.857	12.680	2,383.991	16.876	0.448	Female	53
**105**	117.866	19.286	360.675	5.440	0.809	Male	52
**103**	116.709	34.349	668.226	23.360	0.793	Male	50
**177**	185.529	174.699	770.911	48.421	0.791	Male	59
**202**	2,908.996	132.040	6,253.708	33.533	0.786	Male	57
**591**	77.380	12.795	934.489	11.626	0.761	Male	63
**452**	5,886.027	95.877	9,974.303	13.723	0.740	Male	41
**551**	5,035.737	18.670	596.368	34.794	0.721	Male	61
**38**	68.843	19.788	357.338	1.072	0.710	Male	56
**223**	0.000	15.126	358.596	11.541	0.681	Male	51
**377**	2,110.446	92.937	6,199.594	2.438	0.677	Male	58
**638**	15.399	15.270	842.041	54.896	0.664	Male	56

Table S5a (continued)

Number	AFP (ng/mL)	AFU (mU/mL)	GGT2 (mU/mL)	GPC3 (ng/mL)	HGF (ng/mL)	Gender	Age
**477**	5,622.440	16.328	1,590.350	13.794	0.663	Male	45
**442**	278.796	34.689	1,690.819	9.185	0.655	Male	57
**3**	6,457.829	22.518	748.647	30.870	0.634	Male	35
**99**	3,577.713	14.260	482.519	13.418	0.623	Male	54
**27**	22.148	21.332	759.658	15.382	0.588	Male	49
**557**	476.144	12.537	10,369.306	10.588	0.582	Male	65
**131**	0.000	14.380	601.223	19.254	0.574	Male	59
**479**	21.536	6.344	499.056	12.582	0.568	Male	47
**252**	2,644.511	21.016	1,610.891	2.811	0.556	Male	53
**208**	0.000	8.976	655.398	15.748	0.551	Male	48
**540**	37.661	18.364	12,459.634	2.740	0.545	Male	60
**303**	0.000	20.168	1,204.981	0.169	0.542	Male	62
**14**	63.350	28.140	672.378	3.580	0.538	Male	58
**275**	6,387.175	61.427	8,111.757	10.195	0.522	Male	60
**633**	0.000	16.740	827.658	6.679	0.512	Male	45
**546**	6,887.685	11.580	1,643.010	2.864	0.504	Male	49
**554**	27.681	16.958	9,656.203	9.226	0.499	Male	70
**110**	47.043	17.555	1,416.192	0.837	0.491	Male	54
**159**	0.000	17.724	814.162	9.598	0.480	Male	48
**140**	108.080	21.816	4,109.390	7.904	0.475	Male	60
**413**	5,416.129	49.280	3,943.944	1.226	0.464	Male	53
**651**	25.691	7.664	3,760.865	12.804	0.462	Male	57
**560**	1.731	9.114	612.083	36.287	0.455	Male	61
**55**	5.668	11.730	349.025	0.000	0.445	Male	60
**500**	5.520	6.842	773.137	24.284	0.432	Male	54
**511**	0.000	2.527	828.489	14.507	0.430	Male	47
**601**	10.893	6.794	722.047	12.738	0.426	Male	58
**555**	17.700	15.064	1,704.274	19.228	0.421	Male	67
**460**	427.446	8.722	1,043.711	3.459	0.416	Male	55
**567**	60.284	16.571	697.061	123.702	0.414	Male	52
**649**	0.000	7.224	2,963.970	27.836	0.410	Male	51
**647**	0.000	8.012	1,154.634	18.336	0.404	Male	44
**541**	0.000	14.045	548.059	11.355	0.395	Male	44
**588**	12.766	10.989	667.584	8.292	0.363	Male	82
**609**	67.813	15.849	788.293	5.727	0.362	Male	56

Table S5a (continued)

Number	AFP (ng/mL)	AFU (mU/mL)	GGT2 (mU/mL)	GPC3 (ng/mL)	HGF (ng/mL)	Gender	Age
**527**	0.000	8.462	894.955	33.432	0.354	Male	46
**509**	54.069	0.867	479.415	23.321	0.347	Male	63
**612**	0.000	9.460	845.436	13.573	0.338	Male	59
**372**	567.464	48.923	706.861	2.201	0.317	Male	39
**592**	194.434	23.411	4,312.584	10.272	0.307	Male	57
**584**	10.893	15.404	2,227.369	49.970	0.301	Male	59
**596**	110.155	96.378	20,084.400	19.205	0.299	Male	59
**648**	373.209	6.661	2,776.921	0.000	0.298	Male	66
**613**	0.000	7.990	823.849	5.783	0.289	Male	44
**659**	0.000	11.894	876.212	6.672	0.274	Male	52
**582**	0.000	9.303	847.717	16.436	0.265	Male	69
**583**	0.000	8.099	677.165	70.670	0.263	Male	50
**570**	428.541	5.951	700.031	13.021	0.258	Male	53
**278**	0.000	21.713	1,840.606	2.642	3.730	Male	45
**21**	9,797.067	195.955	1,708.972	355.697	2.633	Male	44
**7**	2.921	60.984	3,854.782	49.639	2.309	Male	50
**190**	9.770	135.316	678.661	20.088	2.238	Male	62
**283**	138.925	75.798	14,314.019	19.238	1.842	Male	40
**59**	7.972	129.682	4,795.568	18.890	1.725	Male	52
**32**	0.000	38.262	1,235.579	0.047	1.511	Male	55
**136**	0.000	51.914	453.519	137.894	1.495	Male	52
**191**	215.151	434.019	742.033	8.891	1.444	Male	55
**58**	6,094.917	97.200	5,485.909	9.234	1.398	Male	66
**70**	6,531.637	105.350	2,163.427	22.357	1.346	Male	60
**197**	6,102.796	156.701	1,691.291	6.805	1.231	Male	43
**79**	0.000	22.205	456.470	73.586	1.217	Male	61
**181**	3,087.239	343.843	406.726	16.245	1.197	Male	72
**34**	9,823.263	115.061	1,487.400	20.222	1.176	Male	38
**139**	102.806	0.000	576.877	3.160	1.141	Male	53
**301**	120.418	76.502	1,234.232	1.998	1.108	Male	56
**114**	1.077	34.375	1,154.684	2.822	1.024	Male	44
**42**	16.655	48.972	505.299	2.167	1.008	Male	50
**85**	71.595	13.032	334.625	2.809	0.968	Male	50
**296**	42.923	99.391	870.927	64.424	0.954	Male	63
**146**	254.268	71.919	4,562.507	12.018	0.945	Male	44

Table S5a (continued)

Number	AFP (ng/mL)	AFU (mU/mL)	GGT2 (mU/mL)	GPC3 (ng/mL)	HGF (ng/mL)	Gender	Age
**212**	0.000	34.467	340.948	45.022	0.935	Male	60
**68**	4,451.586	39.734	4,399.074	18.431	0.926	Male	60
**67**	0.000	18.376	352.272	0.177	0.903	Male	50
**398**	0.000	13.756	3,484.362	32.309	0.900	Male	43
**124**	0.000	46.230	964.002	18.673	0.880	Male	45
**259**	10.536	57.183	1,214.695	26.935	0.854	Male	58
**312**	0.000	25.792	727.271	2.540	0.844	Male	57
**407**	56.071	124.458	3,782.968	18.166	0.837	Male	56
**387**	7.413	86.047	2,383.693	2.254	1.018	Male	56
**563**	161.421	70.705	5,493.725	20.317	0.808	Male	84
**17**	0.000	51.062	3,893.093	42.721	1.033	Male	60
**199**	0.000	45.361	764.494	15.450	0.800	Male	57
**269**	17.476	21.930	351.253	2.608	1.285	Male	78
**173**	858.104	20.280	630.531	16.941	1.299	Male	65
**371**	4,716.357	17.166	1,172.866	1.795	0.428	Male	73
**84**	0.000	14.340	183.371	1.430	0.343	Male	67
**656**	12.794	4.230	3,153.492	4.939	0.266	Male	59

Table S5b The information of 244 hepatitis HCC (validation group)

Number	AFP (ng/mL)	AFU (mU/mL)	GGT2 (mU/mL)	GPC3 (ng/mL)	HGF (ng/mL)	Gender	Age
**650**	9,852.325	12.210	4,283.284	10.591	0.514	Female	44
**47**	9,633.117	18.021	614.191	0.000	0.764	Female	55
**46**	5,382.944	14.497	1,169.839	0.000	0.974	Female	42
**69**	4,984.456	16.111	2,242.602	3.769	0.642	Female	59
**579**	4,560.545	8.460	746.732	5.422	0.270	Female	67
**31**	903.122	155.249	7,233.668	10.753	1.037	Female	57
**537**	554.388	8.258	3,400.518	1.948	0.411	Female	63
**569**	456.925	6.774	713.819	13.728	0.423	Female	62
**587**	318.043	12.313	11,851.811	13.647	0.680	Female	49
**247**	184.034	12.468	529.449	3.014	0.446	Female	65
**610**	129.614	15.537	920.992	10.939	0.352	Female	63
**307**	126.202	8.814	896.832	3.658	1.439	Female	70
**402**	58.073	132.238	15,918.837	14.744	1.003	Female	71
**606**	24.940	6.874	1,219.911	1.542	0.309	Female	57
**566**	19.031	9.298	1,444.578	23.857	0.426	Female	63
**43**	16.655	96.260	226.833	1.354	1.146	Female	56
**575**	9.957	7.336	872.402	7.989	0.285	Female	64
**453**	9.024	10.769	864.200	0.000	0.420	Female	65
**562**	0.000	6.629	6,833.949	5.216	0.347	Female	59
**62**	59.375	60.795	311.097	1.765	2.049	Female	77
**167**	0.000	13.722	1,802.633	4.007	0.574	Female	34
**644**	4,831.734	11.063	4,400.320	5.026	0.718	Female	56
**654**	10,340.953	1.841	657.336	6.432	0.288	Male	54
**306**	9,504.523	83.511	5,806.052	21.382	1.333	Male	49
**615**	7,589.057	17.519	5,724.224	76.724	0.468	Male	56
**82**	6,428.845	32.986	494.283	27.011	0.453	Male	36
**64**	6,373.376	43.353	1,238.855	15.047	0.924	Male	62
**37**	6,232.097	104.771	818.198	1.708	1.259	Male	69
**16**	6,214.831	39.433	875.201	17.254	1.213	Male	45
**273**	6,209.024	20.951	744.541	19.238	1.223	Male	55
**178**	6,136.615	159.149	3,136.487	39.649	0.663	Male	45
**187**	6,071.655	273.419	4,300.544	5.844	0.928	Male	55
**397**	5,999.737	21.141	5,464.136	2.699	0.302	Male	43
**507**	5,575.943	66.806	4,566.809	76.817	0.725	Male	48
**370**	5,285.960	80.587	4,093.100	2.303	0.792	Male	63

Table S5a (continued)

Number	AFP (ng/mL)	AFU (mU/mL)	GGT2 (mU/mL)	GPC3 (ng/mL)	HGF (ng/mL)	Gender	Age
**116**	5,149.800	48.661	1,802.633	2.313	0.460	Male	53
**385**	5,111.872	21.484	2,932.634	7.726	0.788	Male	67
**382**	5,069.321	13.204	766.521	1.535	0.295	Male	65
**180**	4,713.706	703.627	2,962.245	116.129	1.832	Male	46
**475**	4,499.951	6.427	965.083	8.211	0.538	Male	31
**169**	3,518.442	17.517	857.541	5.701	0.667	Male	59
**100**	3,446.153	39.274	666.546	15.214	1.119	Male	59
**388**	2,610.837	19.865	10,162.328	8.240	0.738	Male	56
**489**	2,296.835	11.958	708.857	1.440	0.619	Male	51
**96**	2,195.566	26.766	342.188	13.209	0.843	Male	57
**639**	1,865.760	13.756	674.008	29.225	0.362	Male	27
**577**	1,837.225	20.401	13,565.523	13.708	0.526	Male	50
**78**	1,661.705	10.208	259.838	24.279	0.415	Male	53
**246**	1,553.839	18.211	2,735.734	3.725	0.585	Male	64
**446**	1,372.806	10.520	478.522	1.986	0.365	Male	57
**75**	1,063.240	32.939	830.405	4.104	0.710	Male	45
**461**	733.012	12.567	12,522.107	14.483	0.479	Male	48
**147**	550.388	36.054	1,346.208	18.044	0.906	Male	57
**547**	541.150	10.316	855.376	9.672	0.301	Male	34
**176**	519.243	53.613	569.566	14.721	0.403	Male	53
**576**	477.239	19.977	1,929.334	31.040	0.701	Male	50
**456**	454.474	13.065	1,013.741	0.000	0.464	Male	44
**519**	415.595	12.476	3,443.506	7.593	0.483	Male	51
**317**	404.954	20.603	767.306	21.466	0.945	Male	46
**565**	359.038	10.235	839.661	144.788	0.338	Male	58
**66**	305.264	41.759	1,289.495	2.809	1.075	Male	68
**392**	267.832	30.417	6,689.392	8.830	1.384	Male	58
**201**	260.572	185.869	995.518	60.244	0.566	Male	53
**12**	247.384	11.872	369.806	51.479	0.470	Male	59
**89**	198.840	50.325	593.439	45.623	2.258	Male	44
**184**	192.441	551.973	6,795.512	11.740	0.825	Male	56
**76**	185.720	11.915	144.717	36.082	0.388	Male	58
**53**	178.714	67.456	499.480	2.450	2.363	Male	45
**260**	164.371	33.079	881.132	23.490	0.902	Male	57
**196**	152.945	70.806	0.000	8.925	0.612	Male	65

Table S5a (continued)

Number	AFP (ng/mL)	AFU (mU/mL)	GGT2 (mU/mL)	GPC3 (ng/mL)	HGF (ng/mL)	Gender	Age
**274**	147.021	32.624	4,467.932	3.725	0.570	Male	44
**134**	136.715	34.583	1,371.196	17.293	1.120	Male	62
**573**	136.375	7.276	2,349.475	34.995	0.548	Male	48
**310**	133.141	21.734	737.476	1.321	0.772	Male	48
**593**	95.172	123.940	2,512.192	48.343	0.575	Male	56
**148**	80.199	24.664	804.114	11.727	1.180	Male	62
**653**	67.094	7.128	7,067.529	5.339	0.952	Male	52
**360**	45.236	23.070	474.499	0.508	0.432	Male	56
**261**	32.513	22.376	4,726.772	12.362	0.657	Male	53
**77**	31.877	50.151	256.477	36.130	1.551	Male	55
**81**	25.188	98.377	827.044	33.375	2.118	Male	44
**74**	25.188	17.196	356.473	3.477	0.498	Male	51
**450**	10.025	16.964	610.652	43.078	0.695	Male	45
**585**	9.957	6.734	673.425	18.518	0.262	Male	52
**561**	8.385	14.289	645.260	9.350	0.552	Male	52
**126**	6.352	59.547	6,736.497	48.009	1.509	Male	50
**41**	5.668	16.019	378.950	4.499	0.894	Male	48
**165**	4.845	10.904	539.545	14.631	0.761	Male	56
**534**	4.392	4.815	651.662	4.226	0.298	Male	57
**425**	4.019	15.056	477.629	0.000	0.359	Male	36
**118**	2.584	17.254	805.625	10.396	0.820	Male	54
**594**	0.000	24.314	1,531.477	18.154	3.502	Male	50
**617**	0.000	316.716	15,108.376	30.733	1.689	Male	72
**539**	0.000	74.438	2,992.270	144.788	1.471	Male	54
**88**	0.000	21.376	473.276	8.072	1.009	Male	48
**447**	0.000	56.567	911.517	4.813	0.963	Male	66
**172**	0.000	206.436	649.783	62.461	0.864	Male	63
**614**	0.000	28.599	875.912	10.266	0.844	Male	54
**171**	0.000	20.598	652.352	37.079	0.795	Male	49
**30**	0.000	18.778	766.308	0.000	0.655	Male	55
**455**	0.000	38.916	867.771	0.751	0.624	Male	57
**599**	0.000	17.391	761.693	8.292	0.623	Male	70
**499**	0.000	18.458	895.447	65.053	0.601	Male	62
**162**	0.000	11.712	506.271	8.460	0.544	Male	49
**108**	0.000	17.686	853.619	2.725	0.506	Male	59

Table S5a (continued)

Number	AFP (ng/mL)	AFU (mU/mL)	GGT2 (mU/mL)	GPC3 (ng/mL)	HGF (ng/mL)	Gender	Age
**163**	0.000	11.130	289.584	1.394	0.501	Male	58
**538**	0.000	6.710	425.248	1.602	0.444	Male	63
**396**	0.000	18.430	846.344	2.960	0.405	Male	69
**383**	0.000	3.670	600.391	5.825	0.363	Male	59
**564**	0.000	6.608	382.759	34.513	0.305	Male	66
**658**	0.000	4.340	899.117	0.000	0.266	Male	64
**605**	0.000	8.119	737.756	2.249	0.265	Male	52
**20**	0.000	274.126	3,216.747	3.439	1.822	Male	69
**44**	123.779	121.444	300.813	4.146	0.890	Male	76
**50**	439.658	77.904	633.310	0.153	1.159	Male	55
**130**	70.403	47.763	4,285.802	12.187	1.028	Male	55
**168**	0.000	45.117	931.627	4.128	1.389	Male	61
**611**	0.000	21.927	3,670.746	17.888	0.548	Male	74
**151**	4,520.299	18.982	2,603.688	15.406	0.475	Male	73
**107**	0.000	15.188	722.958	5.798	0.528	Male	53
**619**	7,322.315	12.888	1,191.285	20.746	0.284	Male	61

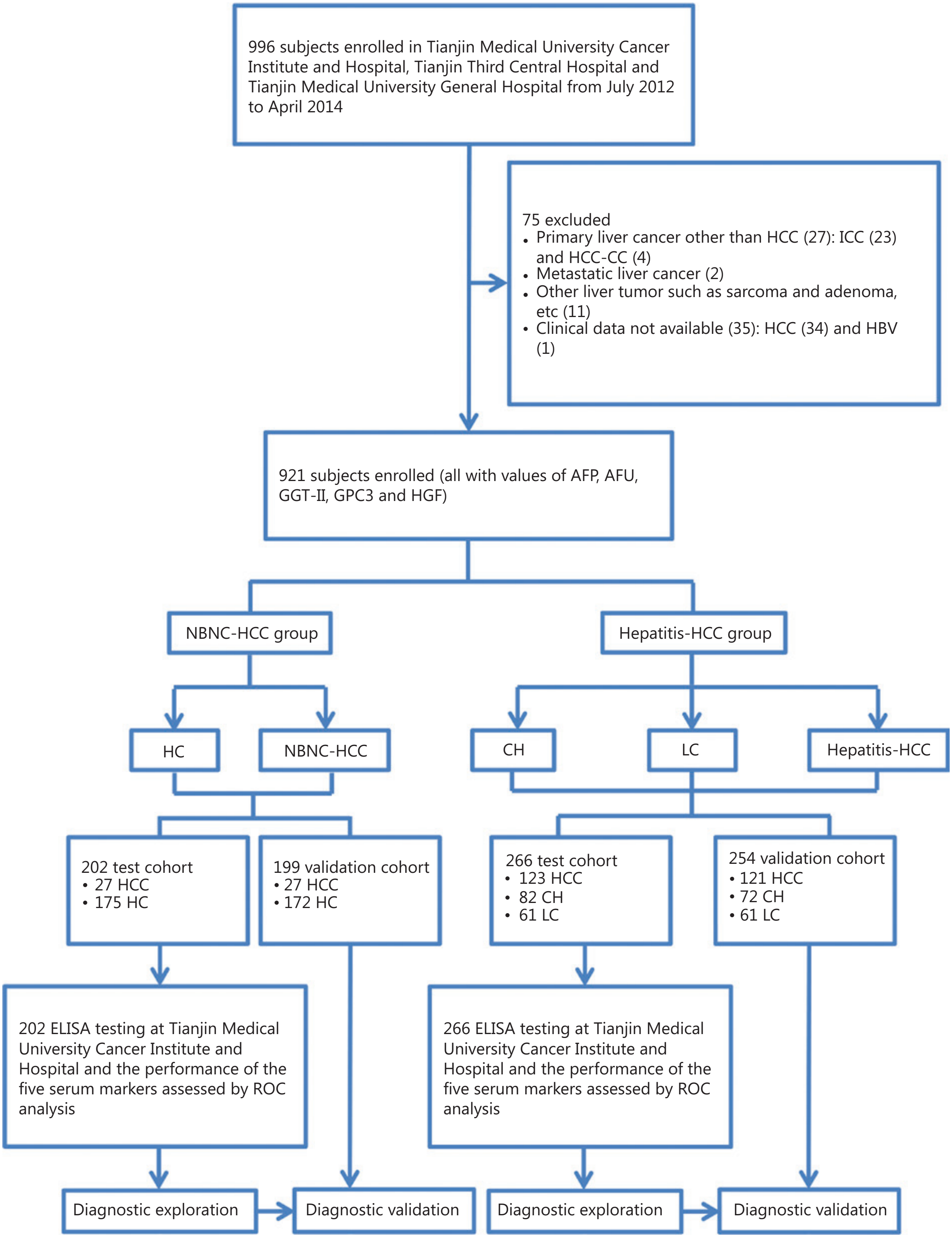

Figure S1 The study design. HC, healthy controls; CH, chronic hepatitis; LC, liver cirrhosis; HCC, hepatocellular carcinoma.

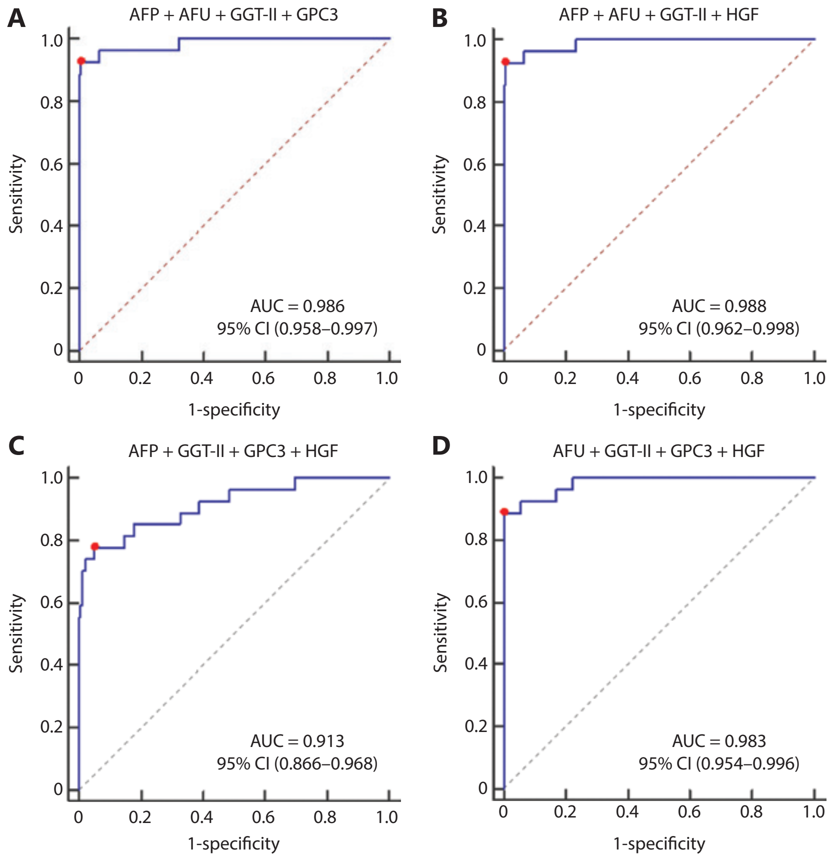

Figure S2 The 4 arrangements of serum biomarkers in the combination model. The sensitivity and specificity represented by the red dots were shown in detail below. (A). AFP + AFU + GGT-II + GPC3 sensitivity: 92.6% and specificity: 99.4%; (B). AFP + AFU + GGT-II + HGF sensitivity: 92.6% and specificity: 98.3%; (C). AFP + GPC3 + GGT-II + HGF sensitivity, 77.8% and specificity: 94.9%; (D). AFU + GPC3 + GGT-II + HGF sensitivity; 88.9% and specificity: 100.0%.

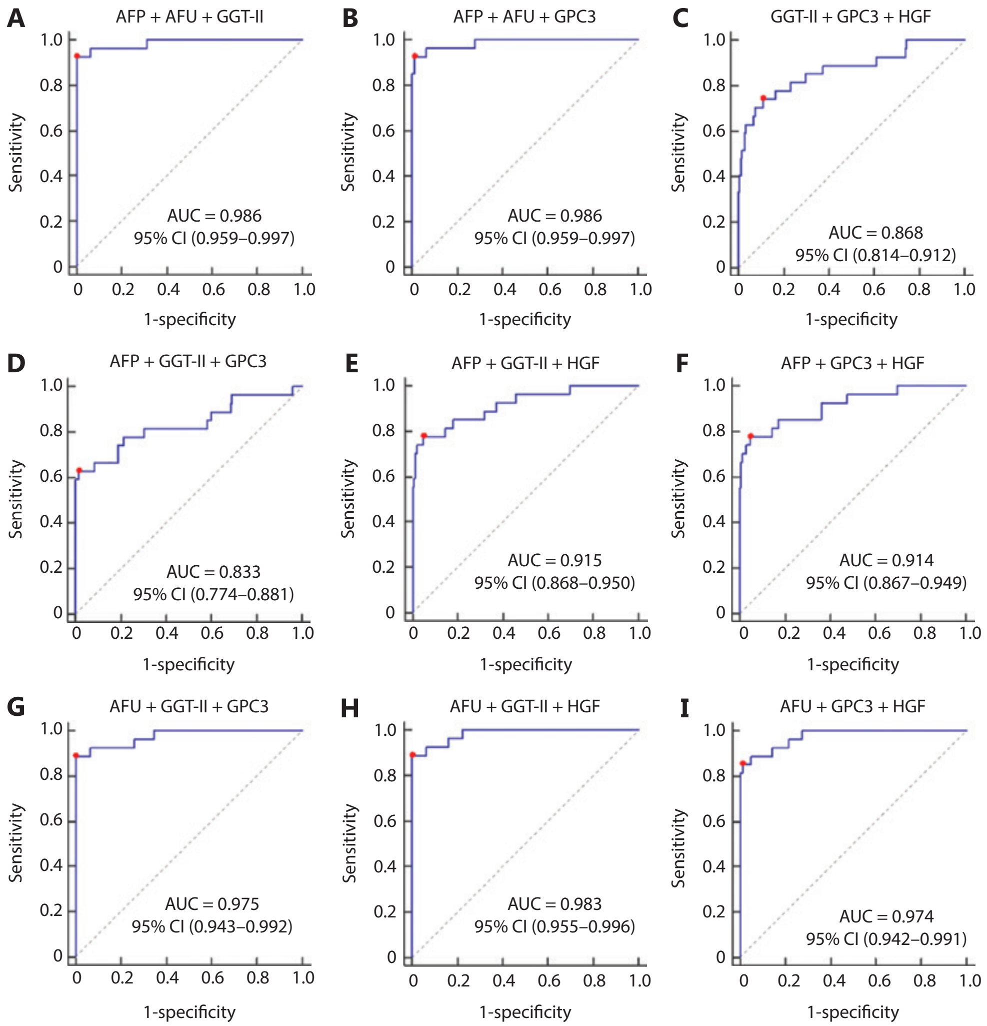

Figure S3 The 3 arrangements of serum biomarkers in the combination model. The sensitivity and specificity represented by the red dots are shown in detail below. (A). AFP + AFU + GGT-II sensitivity: 92.6% and specificity: 100.0%; (B). AFP + AFU + GPC3 sensitivity: 92.6% and specificity: 98.9%; (C). GGT-II + GPC3 + HGF sensitivity: 74.1% and specificity: 89.1%; (D). AFP + GGT-II + GPC3 sensitivity: 63.0% and specificity: 98.3%; (E). AFP + GGT-II + HGF sensitivity: 77.8% and specificity: 94.9%; (F). AFP + GPC3 + HGF sensitivity: 77.8% and specificity: 95.4%; (G). AFU + GGT-II + GPC3 sensitivity: 88.9% and specificity: 100.0%; (H). AFU + GGT-II + HGF sensitivity: 88.9% and specificity: 98.9%; (I). AFU + GPC3 + HGF sensitivity: 85.2% and specificity: 97.7%.

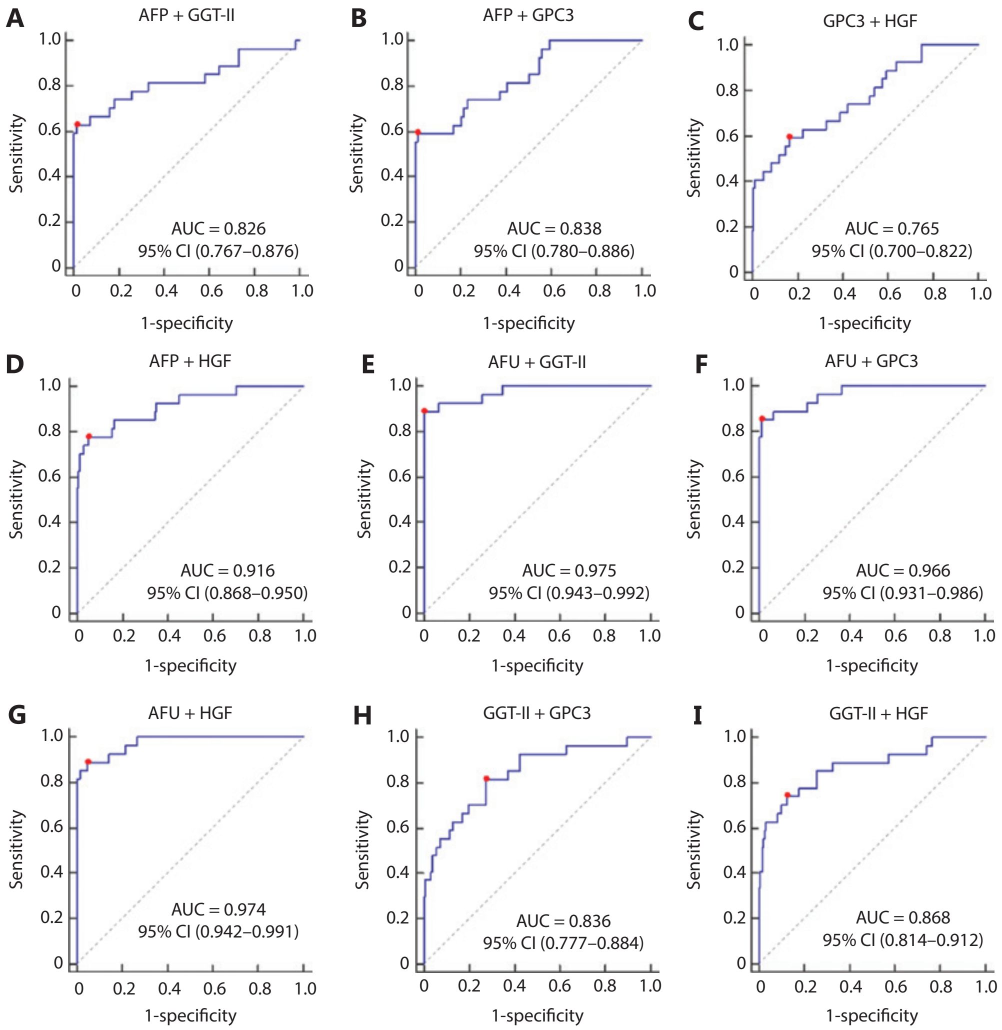

Figure S4 The 2 arrangements of serum biomarkers in the combination model. The sensitivity and specificity represented by the red dots are shown in detail below. (A). AFP + GGT-II sensitivity: 63.0% and specificity: 98.3%; (B). AFP + GPC3 sensitivity: 59.3% and specificity: 98.3%; (C). GPC3 + HGF sensitivity: 59.3% and specificity: 83.4%; (D). AFP + HGF sensitivity: 77.8% and specificity: 94.9%; (E). AFU + GGT-II sensitivity: 88.9% and specificity: 100.0%; (F). AFU + GPC3 sensitivity: 85.2% and specificity: 98.9%; (G). AFU + HGF sensitivity: 88.9% and specificity: 94.9%; (H). GGT-II + GPC3 sensitivity: 81.5% and specificity: 72.5%; (I). GGT-II + HGF sensitivity: 74.1% and specificity: 87.4%.

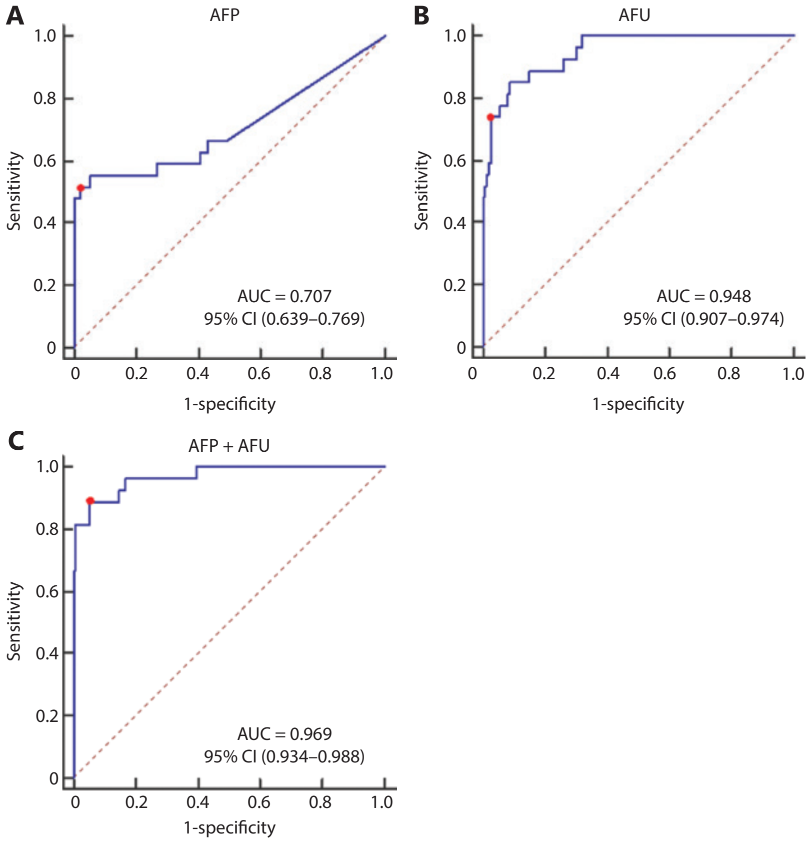

Figure S5 The receiver operating characteristic curves of AFP (A) AFU (B) and AFP + AFU (C) in the detection of the NBNC-HCC validation group. The sensitivity and specificity represented by the red dots are shown in detail below. (A). AFP sensitivity: 51.9% and specificity: 97.7%; (B). AFU sensitivity: 74.1% and specificity: 96.5%; (C). AFP + AFU sensitivity: 88.9% and specificity: 94.8%.

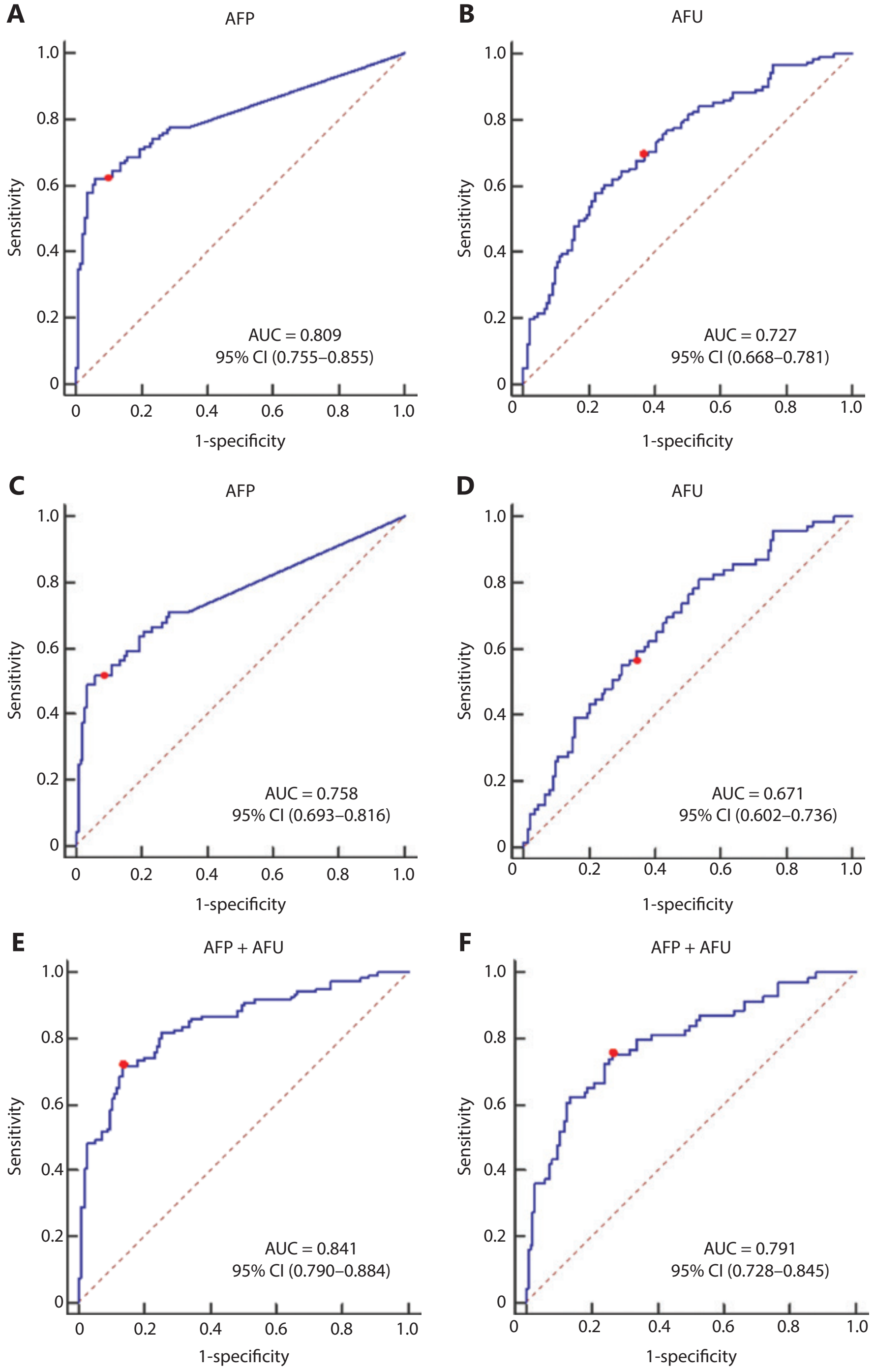

Figure S6 The serum biomarkers AFP, AFU, and combination model of all-stage and early-stage hepatitis-hepatocellular carcinoma (HCC) in the validation cohort. (A), (B), and (E) show the AFP, AFU, and the combination model of all-stage hepatitis-HCC; (C), (D), and (F) show AFP, AFU, and the combination model of early-stage hepatitis-HCC. The sensitivity and specificity represented by the red dots are shown in detail below. (A). AFP sensitivity: 62.8% and specificity: 90.2%; (B). AFU sensitivity: 69.4% and specificity: 65.4%; (C). AFP sensitivity: 52.2% and specificity: 90.2%; (D). AFU sensitivity: 56.5% and specificity: 65.4%; (E). AFP + AFU sensitivity: 71.9% and specificity: 86.5%; (F). AFP + AFU sensitivity: 75.4% and specificity: 73.7%.

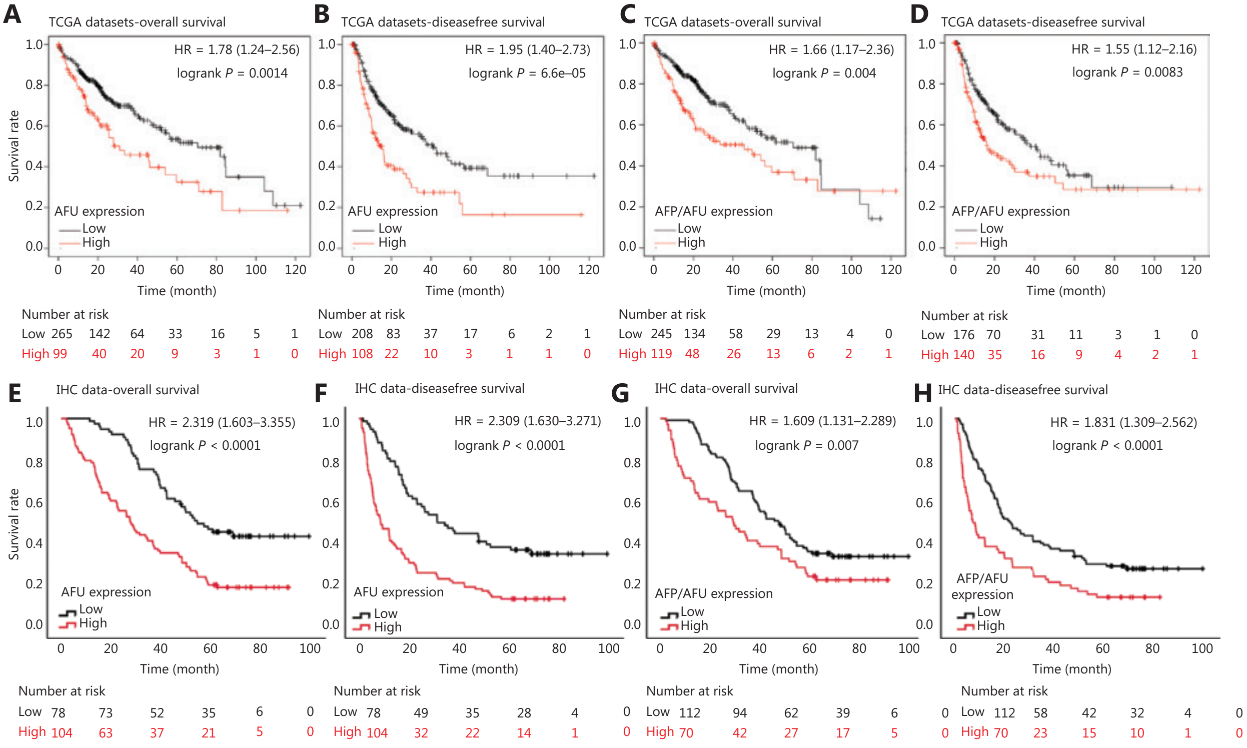

Figure S7 The AFU and AFP/AFU combination model for predicting hepatocellular carcinoma (HCC) prognosis. (A), (B) The survival curves [overall survival (OS) and disease-free survival (DFS)] of HCC patients with different expressions of AFU based on The Cancer Genome Atlas (TCGA) database. (C), (D) The survival curves (OS and DFS) of HCC patients with different expressions of AFP/AFU based on TCGA database. (E), (F) The survival curves (OS and DFS) of HCC patients with different expressions of AFU based on our immunohistochemistry (IHC) data. (G), (H) The survival curves (OS and DFS) of HCC patients with different expressions of AFP/AFU based on our IHC data.
